# TDP-1/TDP-43 Regulates Stress Signaling and Age-Dependent Proteotoxicity in *Caenorhabditis elegans*

**DOI:** 10.1371/journal.pgen.1002806

**Published:** 2012-07-05

**Authors:** Alexandra Vaccaro, Arnaud Tauffenberger, Peter E. A. Ash, Yari Carlomagno, Leonard Petrucelli, J. Alex Parker

**Affiliations:** 1 CRCHUM, Université de Montréal, Montréal, Québec, Canada; 2 Centre of Excellence in Neuromics, Université de Montréal, Montréal, Québec, Canada; 3 Département de Pathologie et Biologie Cellulaire, Université de Montréal, Montréal, Québec, Canada; 4 Department of Neuroscience, Mayo Clinic College of Medicine, Jacksonville, Florida, United States of America; Stanford University School of Medicine, United States of America

## Abstract

TDP-43 is a multifunctional nucleic acid binding protein linked to several neurodegenerative diseases including Amyotrophic Lateral Sclerosis (ALS) and Frontotemporal Dementia. To learn more about the normal biological and abnormal pathological role of this protein, we turned to *Caenorhabditis elegans* and its orthologue TDP-1. We report that TDP-1 functions in the Insulin/IGF pathway to regulate longevity and the oxidative stress response downstream from the forkhead transcription factor DAF-16/FOXO3a. However, although *tdp-1* mutants are stress-sensitive, chronic upregulation of *tdp-1* expression is toxic and decreases lifespan. ALS–associated mutations in TDP-43 or the related RNA binding protein FUS activate the unfolded protein response and generate oxidative stress leading to the *daf-16*–dependent upregulation of *tdp-1* expression with negative effects on neuronal function and lifespan. Consistently, deletion of endogenous *tdp-1* rescues mutant TDP-43 and FUS proteotoxicity in *C. elegans*. These results suggest that chronic induction of wild-type TDP-1/TDP-43 by cellular stress may propagate neurodegeneration and decrease lifespan.

## Author Summary

TAR DNA Binding Protein 43 (TDP-43) is implicated in several human age-dependent neurodegenerative disorders, but until now little was known about TDP-43's role in the aging process. Here we used the nematode *Caenorhabditis elegans* to study the role of the TDP-43 orthologue *tdp-1* in aging and neurodegeneration. In this study we discovered that *tdp-1* is a stress-responsive gene acting within the Insulin/IGF signaling pathway to regulate lifespan and the response to oxidative stress. We found that, although worms missing *tdp-1* were stress-sensitive, elevated expression of *tdp-1* was toxic. We asked if *tdp-1* also responded to the stress caused by toxic proteins found in Amyotrophic Lateral Sclerosis (ALS). Using worm models for ALS, we discovered that mutant TDP-43 generated oxidative stress and induced *tdp-1* expression with negative consequences on neuronal function and lifespan. Consistently, removing *tdp-1* rescued toxicity in our worm ALS models. *tdp-1*'s role in the cellular stress response likely reflects an ancient adaptation to deal with unfavorable environmental conditions that is inappropriately activated and maintained by genetic mutations leading to proteotoxic and oxidative stress. We predict that similar mechanisms may exist in humans, helping explain the involvement of TDP-43 in a growing number of neurodegenerative disorders.

## Introduction

TDP-1 is the *Caenorhabditis elegans* orthologue of the multifunctional DNA/RNA binding protein TDP-43 (TAR DNA Binding Protein 43). Mutations and accumulations of TDP-43 have been found in patients with Amyotrophic Lateral Sclerosis (ALS), Frontotemporal Dementia, and in a growing number of neurodegenerative disorders [1]. As part of its numerous roles in RNA metabolism, TDP-43 is a component of the cytoplasmic ribonucleoprotein complexes known as stress granules that form in response to environmental stresses like heat shock, oxidative and osmotic stress among others [2], [3]. ALS is an age-dependent neurodegenerative disorder and given that TDP-43 is a stress responsive protein we hypothesized that TDP-1 may regulate longevity and the cellular stress response.

In worms a major axis of stress-response signaling and longevity is the Insulin/IGF-signaling (IIS) pathway. IIS follows an evolutionarily conserved and genetically regulated pathway that regulates numerous processes including development, metabolism, fecundity, cellular stress resistance and aging [4]. In *C. elegans*, IIS initiates a phosphorylation cascade through the DAF-2/Insulin-IGF receptor that phosphorylates the forkhead transcription factor DAF-16 and retains it in the cytoplasm [5]–[8]. Hypomorphic *daf-2* mutants relieve DAF-16 phosphorylation causing DAF-16 to translocate to the nucleus where it activates a large number of genes resulting in increased lifespan and augmented stress resistance [9]. *daf-2* mutants are also resistant to numerous stresses including oxidative, osmotic, thermal and proteotoxicity [10]. The IIS pathway likely employs multiple mechanisms to combat a variety of cellular insults but little is known about how these separate functions are regulated by DAF-16.

We attempted to address this issue by asking whether TDP-1 participated in the cellular stress response and longevity pathways in *C. elegans*. We asked if TDP-1 participated in the IIS pathway to specify developmental, stress response, and longevity outcomes. Given the importance of human TDP-43 to neurodegeneration we also asked if *tdp-1* regulated age-dependent proteotoxicity. Our work points to TDP-1 as a key stress response protein at the crossroads of IIS, proteotoxicity and endoplasmic reticulum (ER) stress. Moreover, persistent induction of TDP-1 may actively promote neurodegeneration.

## Results

### *tdp-1* Regulates Lifespan

**Figure 1:**
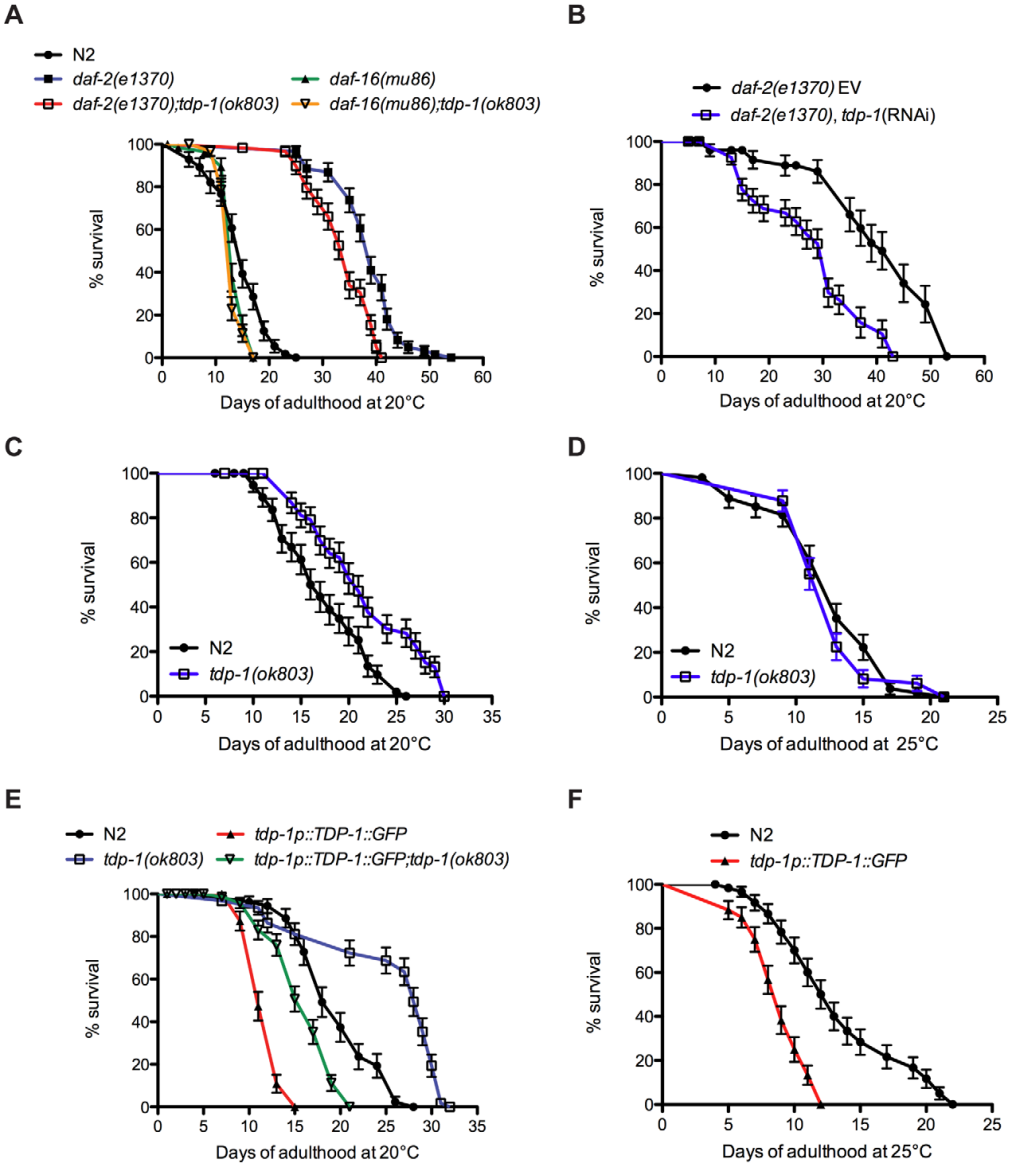
*tdp-1* regulates lifespan. (A) Mutation in *tdp-1* reduced the lifespan of *daf-2(e1370)* mutants but did not further reduce the lifespan of *daf-16(mu86)* mutants. (B) RNAi against *tdp-1* reduced the lifespan of *daf-2(e1370)* mutants. EV is the empty vector control. (C) *tdp-1(ok803)* mutants had increased lifespan compared to N2 worms at 20°C. (D) *tdp-1(ok803)* mutants and N2 worms had comparable lifespan at 25°C. (E) TDP-1::GFP overexpression strains had reduced lifespans compared to N2 worms or *tdp-1(ok803)* mutants at 20°C. (F) TDP-1::GFP transgenic had reduced lifespans compared to N2 worms when grown at 25°C. Please also see Table S1.

Downregulation of DAF-2 extends lifespan and promotes stress resistance via regulation of DAF-16 transcriptional activity [9]. To determine if *tdp-1* participated in the IIS pathway we used a deletion mutant *tdp-1(ok803)* that removes the two RNA Recognition Motifs of TDP-1 (Figure S1A). Looking directly at the IIS pathway and longevity, *daf-2(e1370)* animals were long-lived but this phenotype was significantly reduced in a *daf-2(e1370);tdp-1(ok803)* double mutant strain (Figure 1A, Table S1). Conversely, *daf-16(mu86)* mutants were short-lived compared to wild type worms and the inclusion of *tdp-1(ok803)* did not further reduce *daf-16* mutants' lifespan (Figure 1A, Table S1). We confirmed that *tdp-1* regulated *daf-2* mediated longevity with RNA interference and observed that *tdp-1(RNAi)* reduced the extended lifespan of *daf-2* mutants (Figure 1B, Table S1). These data suggest that *tdp-1* regulates the lifespan phenotypes of reduced IIS and the inability of *tdp-1* to further reduce *daf-16* lifespan suggests that this effect is dependent on *daf-16*.

Surprisingly, despite *tdp-1(ok803)* limiting the extreme lifespan of *daf-2* mutants we observed that at 20°C *tdp-1(ok803)* mutants had a small but significant increase in lifespan versus N2 controls (Figure 1C, Table S1). However this effect was lost when the worms were grown at 25°C (Figure 1D, Table S1) suggesting that *tdp-1* may respond to stressful environmental conditions. Consistently, opposite to reducing *tdp-1* function, overexpression of TDP-1 fused to GFP from the endogenous *tdp-1* promoter (*tdp-1p::TDP-1::GFP*) greatly reduced lifespan compared to wild type N2 worms and *tdp-1* deletion mutants at 20°C (Figure 1E). The lifespan of a *tdp-1(ok803);TDP-1::GFP* strain was greater than *TDP-1::GFP* alone, but less than either N2 worms or the *tdp-1(ok803)* deletion mutant (Figure 1E, Table S1). Finally, TDP-1::GFP transgenics showed a further decrease in mean and maximum lifespan when grown at 25°C compared to N2 worms (Figure 1F, Table S1). These data show that *tdp-1* has a dose-dependent effect on lifespan in worms and that lifespan is sensitive to *tdp-1* expression levels, which is consistent with studies of TDP-43 in flies [11] and mice [12] where overexpression also reduces lifespan.

### *tdp-1* Is Essential for Resistance against Oxidative and Osmotic Stress

Intimately linked to IIS regulation of lifespan in worms is dauer formation and stress resistance [4]. *daf-2(e1370)* mutant larvae show near complete dauer formation when grown at 25°C, but this phenotype was not altered in *daf-2(e1370);tdp-1(ok803)* mutants (Figure S2A) suggesting that *tdp-1* does not function in the dauer formation axis of IIS in worms.

**Figure 2:**
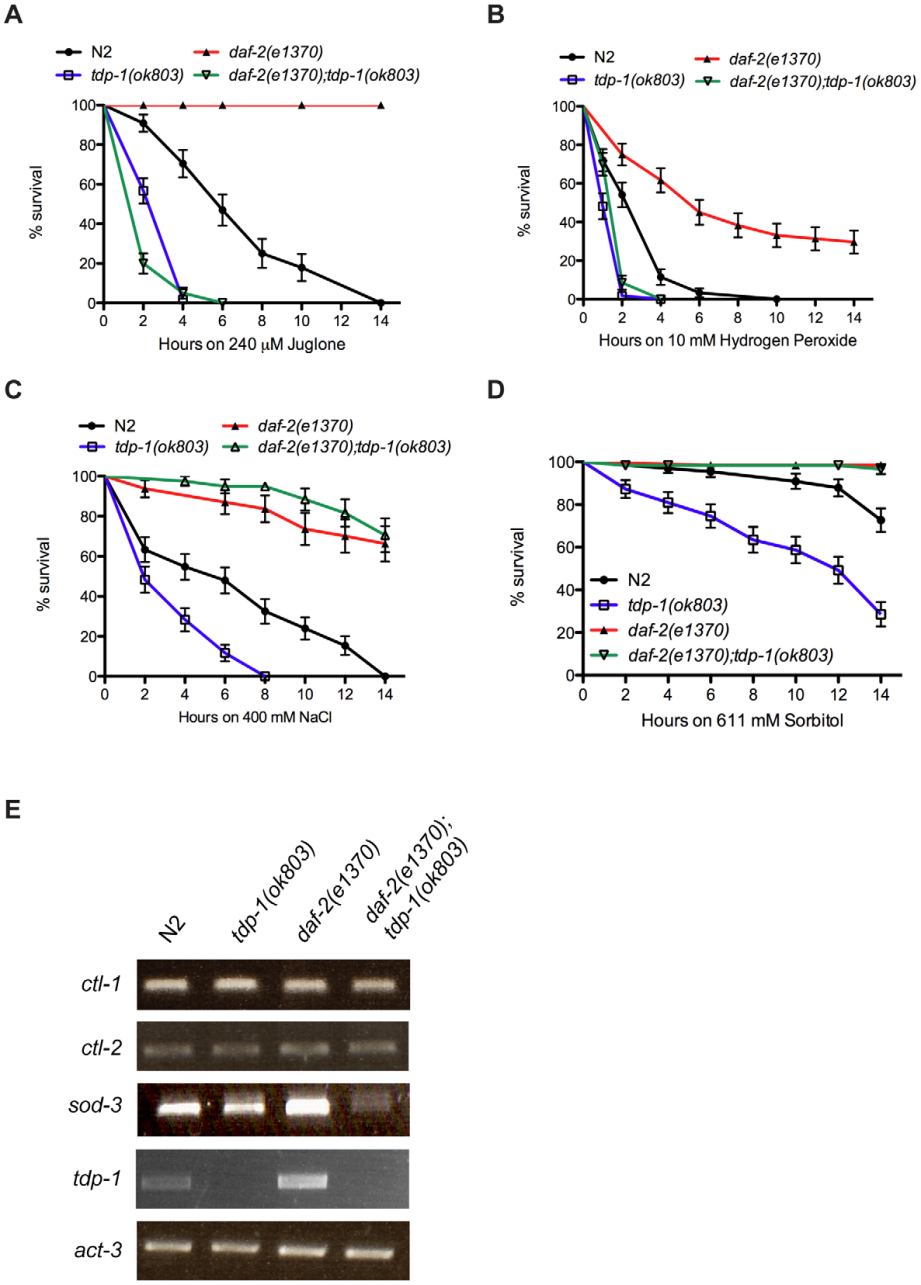
*tdp-1* specifies stress response signaling. (A) *tdp-1(ok803)* mutants were more sensitive to juglone than N2 worms (P<0.001). *daf-2(e1370)* mutants were completely resistant to juglone-induced mortality after 14 hours of exposure. *daf-2(e1370);tdp-1(ok803)* mutants were highly sensitive to juglone toxicity compared to N2 or *daf-2(e1370)* controls (P<0.001). (B) *tdp-1(ok803)* mutants were more sensitive to hydrogen peroxide than N2 worms (P<0.001). *daf-2(e1370)* mutants were more resistant to hydrogen peroxide-induced mortality after 14 hours of exposure than N2 worms (P<0.001). *daf-2(e1370);tdp-1(ok803)* mutants were highly sensitive to hydrogen peroxide toxicity compared to N2 or *daf-2(e1370)* controls (P<0.001). (C) *tdp-1(ok803)* mutants were more sensitive to high NaCl levels than N2 worms (P<0.001), while *daf-2(e1370)* and *daf-2(e1370);tdp-1(ok803)* mutants were more resistant to NaCl-induced mortality than N2 worms after 14 hours of exposure (P<0.001). (D) *tdp-1(ok803)* mutants were more sensitive to high sorbitol levels than N2 worms (P<0.001), while *daf-2(e1370)* and *daf-2(e1370);tdp-1(ok803)* mutants were completely resistant to Sorbitol-induced mortality after 14 hours of exposure (P<0.001). (E) RT-PCR revealed no change in the expression of the catalases *ctl-1* and *ctl-2* by deletion of *tdp-1*. Expression of the superoxide dismutase *sod-3* was diminished in *daf-2(e1370);tdp-1(ok803)* mutants. No expression of *tdp-1* was observed in *tdp-1(ok803)* or *daf-2(e1370);tdp-1(ok803)* mutants. *act-3* was used as an endogenous control. Please see Figure S2 and Table S2.

To further define the role of *tdp-1* in the *in vivo* stress response, we challenged worms against juglone induced oxidative stress. Juglone is a natural product derived from the black walnut tree that raises intracellular oxide levels [13]. Adult wild type N2 worms transferred to juglone plates showed complete mortality after 14 hours, while *daf-2(e1370)* worms were completely resistant (Figure 2A). If *tdp-1* were required for protection against stress we would expect the mutant *tdp-1* worms to be hypersensitive to juglone-induced toxicity. Interestingly, *tdp-1(ok803)* mutants were more sensitive to juglone than N2 worms, and *tdp-1(ok803)* completely abolished *daf-2's* resistance to juglone in the *daf-2(e1370);tdp-1(ok803)* double mutant strain (Figure 2A). To corroborate the role of *tdp-1* in resistance to oxidative stress we used hydrogen peroxide, which is another oxidative stress enhancer. Tested on hydrogen peroxide plates we observed that *tdp-1(ok803)* animals were more sensitive than wild type N2 worms, and *tdp-1(ok803)* sensitized the normally resistant *daf-2* animals to stress-induced mortality (Figure 2B). These data show that *tdp-1* is required for *daf-2* mediated resistance to oxidative stress. Given the effect of *tdp-1(ok803)* on *daf-2* mutants' stress resistance compared to the lack of effect on dauer phenotypes we wondered if *tdp-1* might have a more specific role in the stress response axis of IIS.

Not all forms of stress are equal at the cellular level so we investigated several additional forms of cellular stress including hypertonic stress with elevated sodium chloride (NaCl) or sorbitol, increased temperature, low oxygen and ultraviolet irradiation. Compared to N2 worms, *tdp-1* mutants were sensitive to hypertonic stress from NaCl (Figure 2C). Interestingly, both *daf-2(e1370)* and *daf-2(e1370);tdp-1(ok803)* mutants were resistant to this hypertonic stress suggesting that *tdp-1* may not function, or may function in parallel to the IIS pathway to regulate the response to osmotic stress. To confirm this hypothesis we used sorbitol, another hypertonic stress producer, and similarly observed that *tdp-1(ok803)* mutants were sensitive to osmotic stress but *daf-2*(e1370) and *daf-2(e1370);tdp-1(ok803)* mutants were both resistant after either 14 hours (Figure 2D) or 48 hours of exposure (Figure S2B). Finally, *tdp-1* had no discernable role in the response to thermal stress, hypoxia, or radiation since there was no difference between N2 and *tdp-1(ok803)* worms, as well as *daf-2(e1370)* and *daf-2(e1370);tdp-1(ok803)* mutants in response to these stresses (Figure S2C–S2E). Thus, *tdp-1* functions in the IIS pathway to specify the response to oxidative stress and lifespan, but *tdp-1* is dispensable for the regulation of hypertonic stress by the IIS pathway. A second *tdp-1* deletion allele, *ok781*, was available for investigation and although we observed that *tdp-1(ok781*) animals had a modest increase in lifespan, they were statistically indistinguishable from wild type N2 worms in stress assays (Figure S3).

A component of DAF-2's response to cellular stress is induction of DAF-16 stress response genes [9]. Thus we examined if DAF-16 targets implicated in oxidative stress signaling were affected by *tdp-1*. We used RT-PCR to examine expression levels of three DAF-16 genes including the catalases *ctl-1* and *ctl-2*, as well as the superoxide dismutase *sod-3* in various genetic backgrounds. First of all, no expression of *tdp-1* was observed in strains containing the deletion allele *tdp-1(ok803)* (Figure 2E). Next we observed that *ctl-1* and *clt-2* expression levels were unaltered by the deletion of *tdp-1*, either alone or in combination with *daf-2(e1370)* (Figure 2E). However, we observed that *sod-3* expression was greatly reduced in *daf-2(e1370);tdp-1(ok803)* animals (Figure 2E). These data suggest that under low IIS conditions *tdp-1* is required for the expression of certain DAF-16 targets implicated in oxidative stress and may partially explain the sensitivity of *tdp-1* mutants to juglone and hydrogen peroxide.

### Stress and Insulin/IGF Signaling Induce TDP-1 Expression

**Figure 3:**
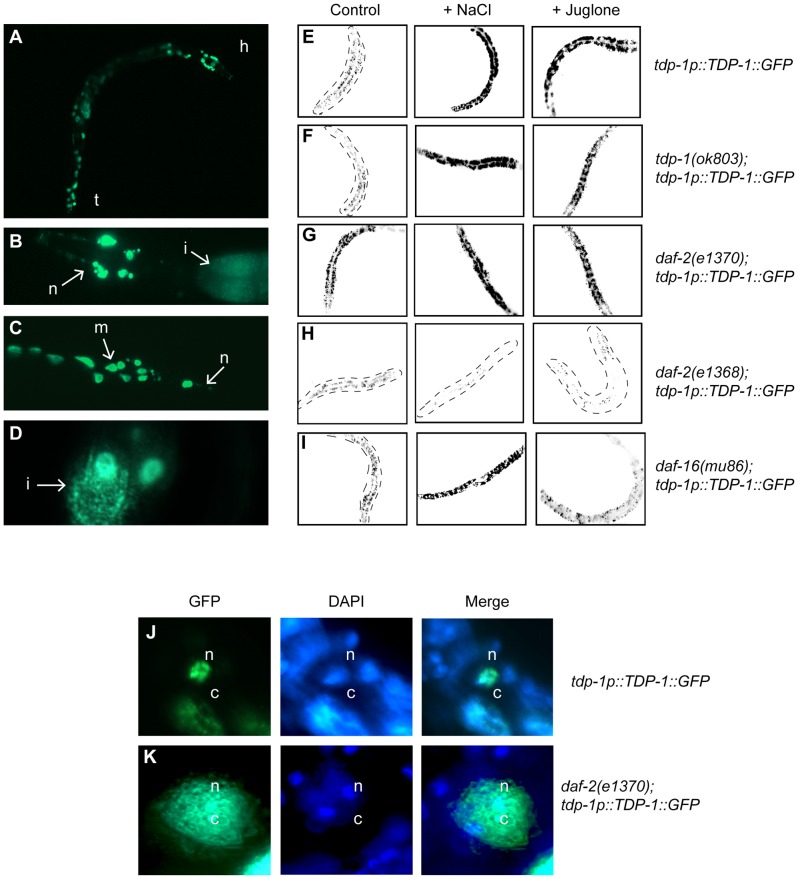
Insulin/IGF signaling and cellular stress regulate TDP-1 expression. (A) Fluorescent signals observed under low magnification in an adult transgenic worm expressing a full-length TDP-1::GFP fusion under control of the endogenous *tdp-1* promoter. The head (h) and tail (t) of the animal are indicated. GFP is observed in many tissues and appears to be primarily nuclear. (B) High magnification image of the head of a TDP-1::GFP transgenic revealing expression in head neurons (n) as well as the intestine (i). (C) High magnification image of the tail region of a TDP-1::GFP transgenic revealing expression in tail neurons (n) as well as muscle cells (m). (D) High magnification image revealing the expression of TDP-1::GFP in intestinal (i) cells. (E–I) are low-resolution photographs of young adult TDP-1::GFP transgenic worms. The images have been taken at the same exposure, converted to black and white and photo-reversed to aid visualization. Difficult to see worms are outlined. (E) TDP-1::GFP is widely expressed and primarily nuclear under normal conditions. TDP-1::GFP expression was highly induced by osmotic stress with NaCl or oxidative stress with juglone compared to untreated worms as seen in the left panel. (F) TDP-1::GFP expression was observed in *tdp-1(ok803)* mutants and was induced by osmotic and oxidative stress. (G) Compared to untreated controls in (A) TDP-1::GFP expression was elevated in *daf-2(e1370)* mutants and further upregulated by stress conditions. (H) TDP-1::GFP remained lowly expressed in *daf-2(e1368)* mutants under normal and stress conditions. (I) TDP-1::GFP showed faint expression and nuclear localization in *daf-16(mu86)* mutants. Osmotic stress induced TDP-1::GFP expression in *daf-16(mu86)* mutants. TDP-1::GFP was not induced by oxidative stress in *daf-16(mu86)* mutants. (J) TDP-1::GFP is primarily expressed in the nuclei (n, marked with DAPI) of non-stressed transgenics. (K) TDP-1::GFP expression is increased and is now also found in the cytoplasm (c) of *daf-2* animals. Please also see Figure S4.

Given the remarkable sensitivity of *tdp-1(ok803)* mutants to oxidative and osmotic stresses we next wanted to see if there were *in vivo* changes in TDP-1 expression in response to these two stress conditions. In wild type unstressed animals TDP-1::GFP is lowly expressed (Figure 3A), primarily nuclear and is expressed in most tissues (Figure 3B–3D). We next tested if shifting TDP-1::GFP animals to either NaCl or juglone plates affected TDP-1::GFP expression. Young adult TDP-1::GFP worms were exposed to either NaCl or juglone for 90 minutes and live animals were examined for changes in TDP-1::GFP expression. Strikingly, exposure to hypertonic or oxidative stress greatly increased TDP-1::GFP expression compared to untreated control worms as detected by visual inspection and quantification of images (Figure 3E and Figure S4A). Endogenous *tdp-1* was not required for expression of the TDP-1::GFP transgene since expression was induced by stress in *tdp-1(ok803)* deletion mutants (Figure 3F and Figure S4B). As a control for generic effects of stress on transgene expression we tested two other strains and observed no induction with a neuronal GFP reporter, while juglone and NaCl induced expression of the well-characterized *sod-3p::GFP* reporter (Figure S5).

Having shown that our TDP-1::GFP transgenics are potent stress reporters, using *daf-2* and *daf-16* mutants we tested if IIS regulated this effect. We observed that TDP-1::GFP was highly induced in the *daf-2(e1370);TDP-1::GFP* strain (Figure 3G) compared to TDP-1::GFP controls (Figure 3A) and was further elevated in *daf-2(e1370)* mutants exposed to stress (Figure 3G and Figure S4C). Given the pleiotropic phenotypic effects of *daf-2* mutations we tested another allele and observed that opposite to *e1370*, TDP-1::GFP expression remained low under normal and stress conditions in the presence of the *daf-2(e1368)* mutation (Figure 3H and Figure S4C) suggesting a complex role for IIS in TDP-1 expression.

Looking deeper into the IIS pathway, previous research has shown that the increased stress resistance of *daf-2* mutants is dependent on *daf-16* mediated nuclear transcription [14]. TDP-1::GFP was not upregulated by mutation in *daf-16* under normal conditions (Figure 3I). Next, to directly test if *daf-16* was essential for the stress dependent induction of *tdp-1* we exposed *daf-16(mu86);TDP-1::GFP* transgenics to stress. Exposure to NaCl continued to induce TDP-1::GFP expression in *daf-16* mutants (Figure 3I) while treatment with juglone failed to induce TDP-1::GFP expression in the *daf-16* mutants (Figure 3I and Figure S4D). These data suggest that the expression of TDP-1 in response to oxidative stress is dependent on *daf-16*, while induction of TDP-1 by osmotic stress is independent, which is consistent with our data showing that *tdp-1* is not required for *daf-2*'s resistance to hypertonic stress (Figure 2C and 2D). Additionally, the upregulation of TDP-1::GFP in *daf-2(e1370)* mutants was abolished by *daf-16*(RNAi) directly linking the IIS pathway to *tdp-1* expression (Figure S6A and S6B, and Figure S4E). Finally, stress resistance and lifespan phenotypes from low IIS requires nuclear localization of DAF-16 and transcriptional activation of target genes [6], [9], [14] but we observed that *tdp-1(ok803)* had no effect on DAF-16::GFP stress-induced nuclear localization (Figure S6C–S6F).

Since TDP-43 is known to shuttle between the nucleus and cytoplasm we wondered if the subcellular distribution of TDP-1::GFP was altered under stress conditions. Examining TDP-1::GFP animals under high magnification we observed that when exposed low IIS from the *daf-2(e1370)* mutation TDP-1::GFP was no longer restricted to the nucleus and was observed in the cytoplasm (Figure 3J and 3K). These data suggest stress and/or low IIS significantly influences the expression and cellular distribution of TDP-1 proteins.

### Endogenous TDP-1 Is Induced by Stress

**Figure 4:**
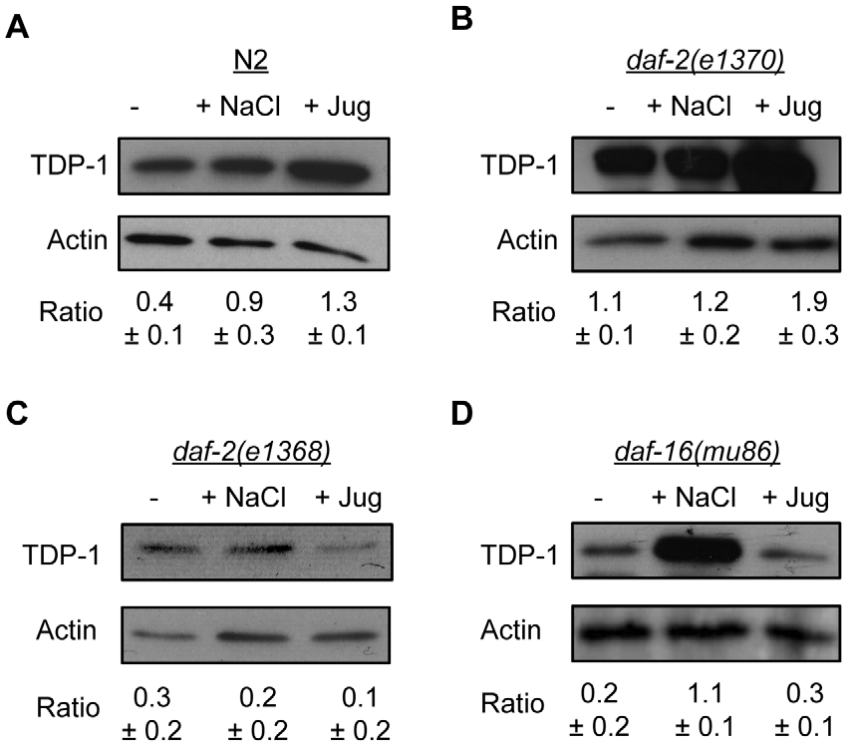
Endogenous TDP-1 protein levels influenced by stress and Insulin/IGF signaling. Western blotting of worm extracts from different strains under normal and stress conditions with an anti-TDP-1 antibody. (A) Osmotic and oxidative stress significantly increased the expression of TDP-1 in N2 worms. (B) The level of TDP-1 protein was comparable in untreated and osmotically stressed *daf-2(e1370)* worms. TDP-1 protein expression was significantly increased by exposure to oxidative stress in *daf-2(e1370)* worms. (C) TDP-1 protein levels remained low in *daf-2(e1368)* animals with or without stress. (D) TDP-1 protein expression was significantly increased by osmotic stress in *daf-16(mu86)* animals. Oxidative stress did not induce TDP-1 expression in *daf-16* mutants.

To make certain that the induction of TDP-1 by stress was not a phenomenon restricted to our transgenic reporter strain we examined endogenous TDP-1 protein levels under normal and stress conditions. Using a monoclonal antibody against *C. elegans* TDP-1 (Figure S7) and western blotting we observed a significant increase in TDP-1 protein levels in N2 worms exposed to hyperosmotic or oxidative stress compared to untreated controls (Figure 4A). To confirm the opposing effects of *daf-2* mutations on TDP-1 expression we examined *daf-2(e1370)* and *daf-2(e1368)* mutants under normal and stressed conditions. As seen with our TDP-1::GFP reporter strain, we observed that TDP-1 protein levels were elevated in *daf-2(e1370)* animals, and we observed a further increase in TDP-1 levels in *daf-2(e1370)* animals exposed to oxidative stress (Figure 4B). Consistent with what we observed in the *daf-2(e1368)*;TDP-1::GFP animals, we observed that TDP-1 protein levels remained low in *daf-2(e1368)* animals under normal and stress conditions (Figure 4C). Finally, endogenous TDP-1 protein was lowly expressed in *daf-16* mutants, greatly increased upon exposure to osmotic stress, but again remained low in *daf-16* animals treated with juglone (Figure 4D) in agreement with our findings with the *daf-16(mu86)*;TDP-1::GFP reporter strain. In summary, TDP-1 is a stress responsive protein whose expression is greatly influenced by the IIS pathway especially in the context of oxidative stress.

### Proteotoxicity Induces TDP-1 Expression

Our findings begin to outline a complex role for TDP-1 in lifespan and the cellular stress response in relation to the IIS pathway. As one of our main interests is understanding the role of aging and stress signaling in the context of age-dependent neurodegeneration we next investigated how proteotoxicity contributed to these processes.

**Figure 5:**
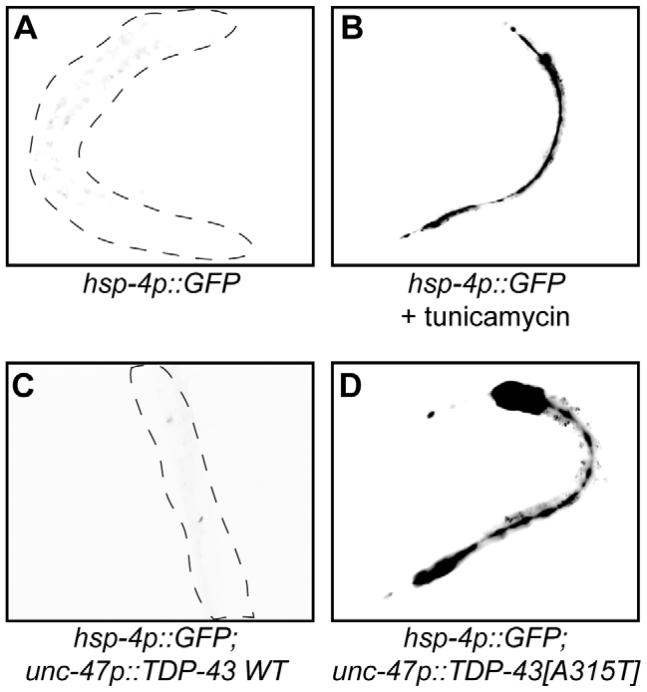
Mutant TDP-43 induces HSP-4/BiP expression. (A to D) are low-resolution photographs of young adult transgenic worms. The images have been converted to black and white and photo-reversed to aid visualization. Difficult to see worms are outlined. (A) Representative image of a young adult worm containing an integrated *hsp-4p::GFP* transgene under non-stressed conditions. (B) *hsp-4p::GFP* expression was induced by the chemical ER stressor tunicamycin. (C) *hsp-4p::GFP* expression was not induced by wild type TDP-43 in a double transgenic *TDP-43 WT;hsp-4p::GFP* strain. (D) *hsp-4p::GFP* expression was induced by mutant TDP-43 in a double transgenic *TDP-43[A315T];hsp-4p::GFP* strain.

A feature of many late neurodegenerative disorders is proteotoxic stress caused by misfolded proteins and mutations in human TDP-43 are believed to cause proteotoxicity leading to neuronal dysfunction and cell death [15]. We examined this directly with transgenic worm strains expressing human wild type and mutant TDP-43 in worm motor neurons [16]. HSP-4 is a *C. elegans* Hsp70 protein orthologous to mammalian Grp78/BiP, and the transgenic *C. elegans hsp-4p::GFP* reporter is activated in response to misfolded proteins within the endoplasmic reticulum (ER), including chemically by compounds like tunicamycin (Figure 5A and 5B) [17]. Using this reporter we observed that transgenic strains expressing wild type TDP-43 did not induce reporter expression whereas transgenics expressing mutant TDP-43 strongly induced *hsp-4p::GFP* expression (Figure 5C and 5D). These data indicate that mutant TDP-43 toxicity may activate the ER unfolded protein response (UPR^ER^). We did not observe induction of GFP in reporter strains for the mitochondrial chaperones *hsp-6*/Hsp70 and *hsp-60*/Hsp10/60 or the cytoplasmic chaperone *hsp-16.2*/Hsp16 (Figure S8).

**Figure 6:**
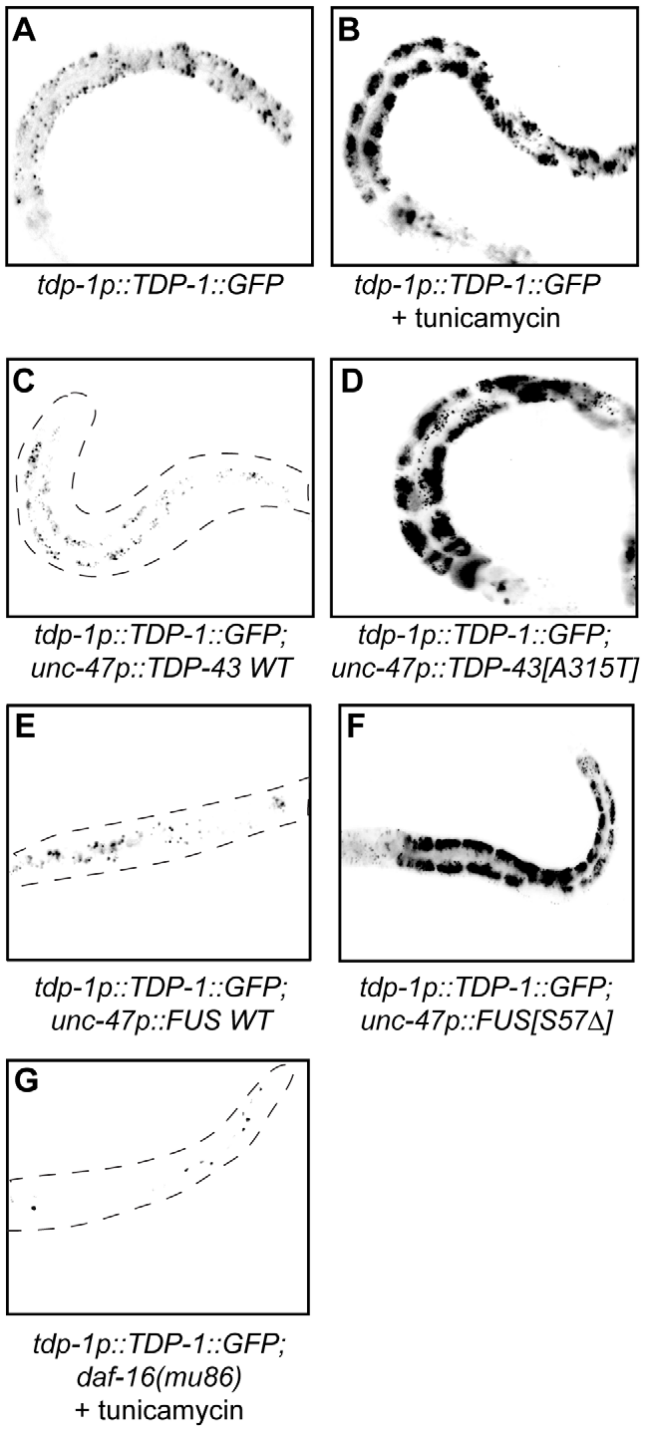
TDP-1 expression is induced by proteotoxicity. (A–G) are low-resolution photographs of young adult transgenic worms. The images have been converted to black and white and photo-reversed to aid visualization. Difficult to see worms are outlined. (A) Low expression and nuclear localization of TDP-1::GFP under normal growth conditions. (B) The ER stressor tunicamycin highly induced TDP-1::GFP expression. (C) Wild type TDP-43 did not induce TDP-1::GFP expression in a *TDP-1::GFP*;*TDP-43 WT* transgenic strain. (D) Mutant TDP-43 induced TDP-1::GFP expression in a *TDP-1::GFP;TDP-43[A315T]* transgenic strain. (E) Wild type FUS did not induce TDP-1::GFP expression in a *TDP-1::GFP*;*FUS WT* transgenic strain. (F) Mutant FUS induced TDP-1::GFP expression in a *TDP-1::GFP;FUS[S57Δ]* transgenic strain. (G) Mutation in *daf-16* blocked the induction of TDP-1::GFP expression by tunicamycin treatment. Please also see Figure S4.

Since we identified TDP-1 as a stress responsive protein we wondered if it also responded to ER and proteotoxic stress. We noticed increased expression of TDP-1::GFP in worms grown on plates with the ER stress inducing compound tunicamycin compared to untreated controls (Figure 6A and 6B). Looking at proteotoxic stress with our TDP-43 transgenics we observed that wild type TDP-43 had no effect on TDP-1::GFP expression while mutant TDP-43 strongly induced TDP-1 expression (Figure 6C and 6D, and Figure S4F). To confirm that induction of TDP-1::GFP was due to protein misfolding and proteotoxicity and not to an artifact of mutant TDP-43 transgenes we examined another proteotoxicity model based on the expression of wild type and mutant human FUS in motor neurons [16]. FUS is a nucleic acid binding protein related to TDP-43 that has also been implicated in ALS and dementia and the expression of an ALS-linked FUS allele in *C. elegans* motor neurons produces degenerative phenotypes similar to mutant TDP-43 [16]. Similar to the TDP-43 model, we observed no effect on TDP-1 expression from wild type FUS but mutant FUS greatly induced TDP-1 expression (Figure 6E and 6F, and Figure S4F). These data suggest that misfolded mutant TDP-43 and FUS initiate the UPR^ER^, which in turn activates expression of TDP-1. Finally, activation of TDP-1 by ER stress converged on the IIS since induction of TDP-1::GFP expression by tunicamycin was blocked by a null mutation in *daf-16* (Figure 6G).

### Oxidative Stress Links Mutant TDP-43 and FUS Proteotoxicity to DAF-16

**Figure 7:**
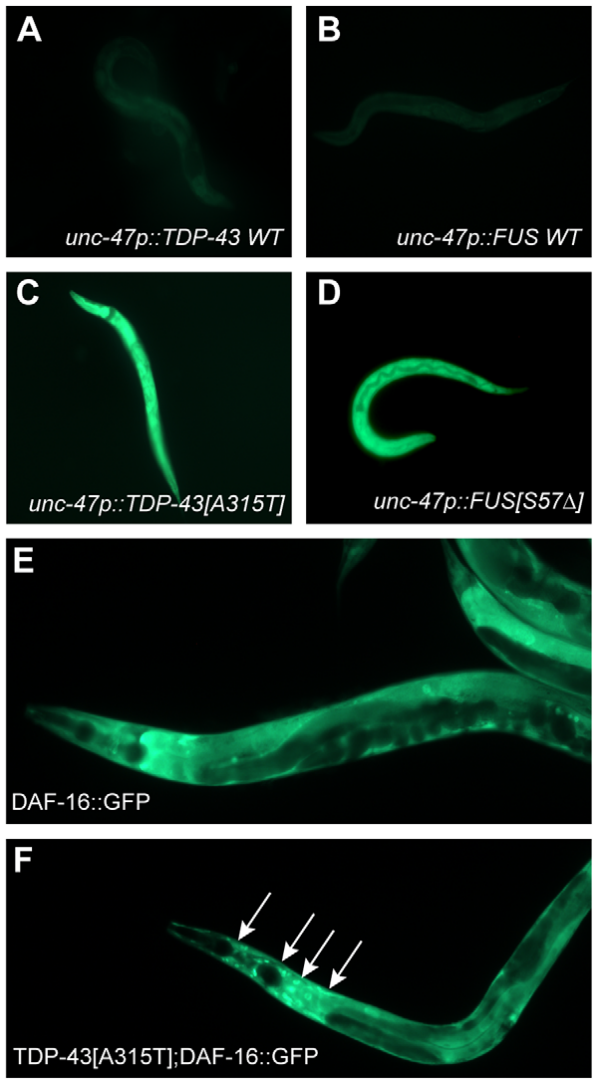
Mutant TDP-43 and FUS increase oxidative stress. (A–D) are low-resolution images of young adult transgenic worms stained with dihydrofluorescein diacetate. Transgenics expressing wild type (A) TDP-43 or (B) wild type FUS showed no fluorescence compared to the bright fluorescent signals observed in animals expressing (C) mutant TDP-43 or (D) mutant FUS. (E to F) are higher magnification images of DAF-16::GFP transgenics. (E) Diffuse appearance of predominantly cytoplasmic DAF-16::GFP. (F) Introduction of mutant TDP-43 causes nuclear localization (arrows) of DAF-16::GFP.

A consequence of processing misfolded proteins within the ER is the production of reactive oxygen species as part of what is termed the integrated stress response [18]. Our data show that *tdp-1* responds to oxidative stress in a *daf-16* dependent matter so we hypothesized that the ER stress produced from mutant TDP-43 and FUS may generate oxidative stress thus linking proteotoxicity to the IIS pathway. To test this hypothesis we stained our transgenic TDP-43 and FUS strains with dihydrofluorescein diacetate (DHF), a compound that fluoresces when exposed to intracellular peroxides associated with oxidative stress [18]. We observed no DHF signal from wild type TDP-43 and FUS transgenics but strong fluorescence from mutant TDP-43 and transgenics (Figure 7A–7D). These data suggest that in addition to activating the UPR^ER^ mutant TDP-43 and FUS generate oxidative stress. To further establish the link between proteotoxicity and the IIS we examined the subcellular localization of DAF-16. DAF-16::GFP is typically cytoplasmic under non-stressed and/or low insulin signaling conditions (Figure 7E). Consistently we observed nuclear localization of DAF-16 in a TDP-43[A315T];DAF-16::GFP strain indicating that mutant TDP-43 causes cellular stress that is transmitted by the IIS pathway (Figure 7F).

### Opposing Effects of DAF-2 on Proteotoxicity

**Figure 8:**
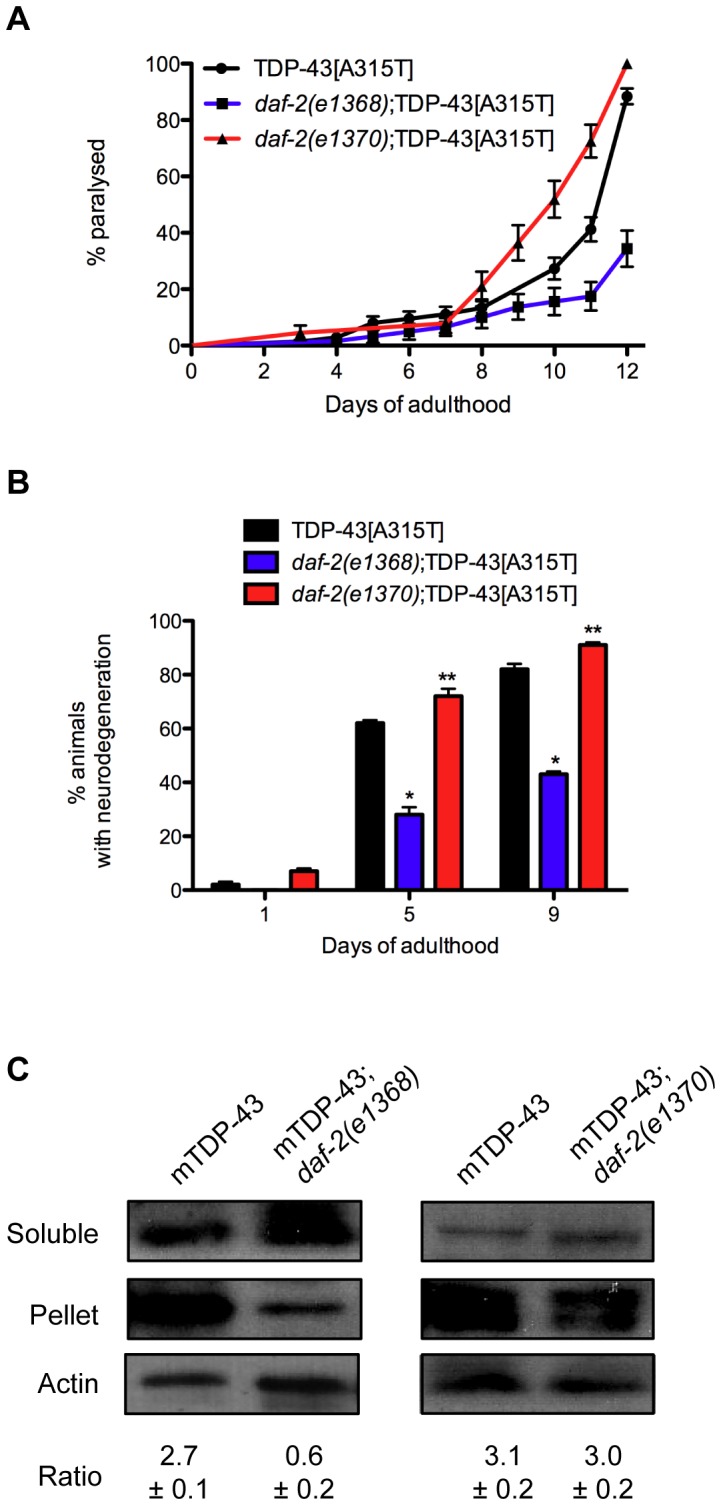
Opposing effects of *daf-2* on proteotoxicity. *(A) daf-2(e1368)*;TDP-43[A315T] animals had significantly deceased rates of paralysis, while *daf-2(e1370)*;TDP-43[A315T] animals had enhanced paralysis compared to TDP-43[A315T] transgenics alone (P<0.001). (B) *daf-2(e1368)*;TDP-43[A315T] animals had significantly deceased motor neuron degeneration, while *daf-2(e1370)*;TDP-43[A315T] animals had enhanced neurodegeneration compared to TDP-43[A315T] transgenics alone (*P<0.001, **P<0.05). (C) Western blotting revealed a significant reduction in the amount of insoluble TDP-43 protein in extracts from *daf-2(e1368)*;TDP-43[A315T] animals compared to TDP-43 alone. *daf-2(e1370)* had no effect on mutant TDP-43 solubility. Please also see Table S3.

Several studies have shown that reduced *daf-2* function suppresses proteotoxicity [19]–[22]. However we found that *daf-2* mutations have opposite effects on TDP-1 expression and if elevated TDP-1 expression were cytotoxic then we would expect to see opposing effects of *daf-2(e1368)* and *daf-2(e1370)* on TDP-43 toxicity in our models. To examine this directly we created *daf-2(e1368)*;TDP-43[A315T] and *daf-2(e1370)*;TDP-43[A315T] strains and scored paralysis phenotypes. Interestingly, we observed that *daf-2(e1368)* suppressed paralysis while *daf-2(e1370)* enhanced paralysis compared to TDP-43[A315T] alone (Figure 8A). This intriguing finding suggests that *daf-2* can have variable effects on proteotoxicity. Furthermore, *daf-2(e1368)* significantly reduced the motor neuron degeneration caused by mutant TDP-43 [16] while *daf-2(e1370)* enhanced degeneration (Figure 8B). Since the IIS pathway is believed to regulate the expression of numerous protein quality control genes we examined if the two *daf-2* alleles had an effect on misfolded mutant TDP-43 with a biochemical assay to detect protein aggregation. Here, homogenized protein extracts from transgenic worms are separated into two fractions, supernatant (detergent-soluble) and pellet (detergent-insoluble) and by western blotting with human TDP-43 antibodies we have previously shown that mutant TDP-43 is prone to aggregation and is highly insoluble [16]. Looking at protein extracts from TDP-43[A315T], *daf-2(e1368)*;TDP-43[A315T], and *daf-2(e1370)*;TDP-43[A315T] animals we observed that *daf-2(1368)* greatly reduced the amount of insoluble TDP-43 compared to control transgenics while *daf-2(e1370)* had no effect (Figure 8C). In summary these data suggest that different *daf-2* alleles have widely variable effects on proteotoxicity but are consistent for each allele: *e1368* reduces mutant TDP-43 insolubility, suppresses mutant TDP-43 induced paralysis, neurodegeneration, and keeps TDP-1 expression low while *e1370* has no effect on protein insolubility, enhances paralysis, neurodegeneration and induces TDP-1 expression.

### TDP-1 Regulates Mutant TDP-43 and FUS Neuronal Toxicity

**Figure 9:**
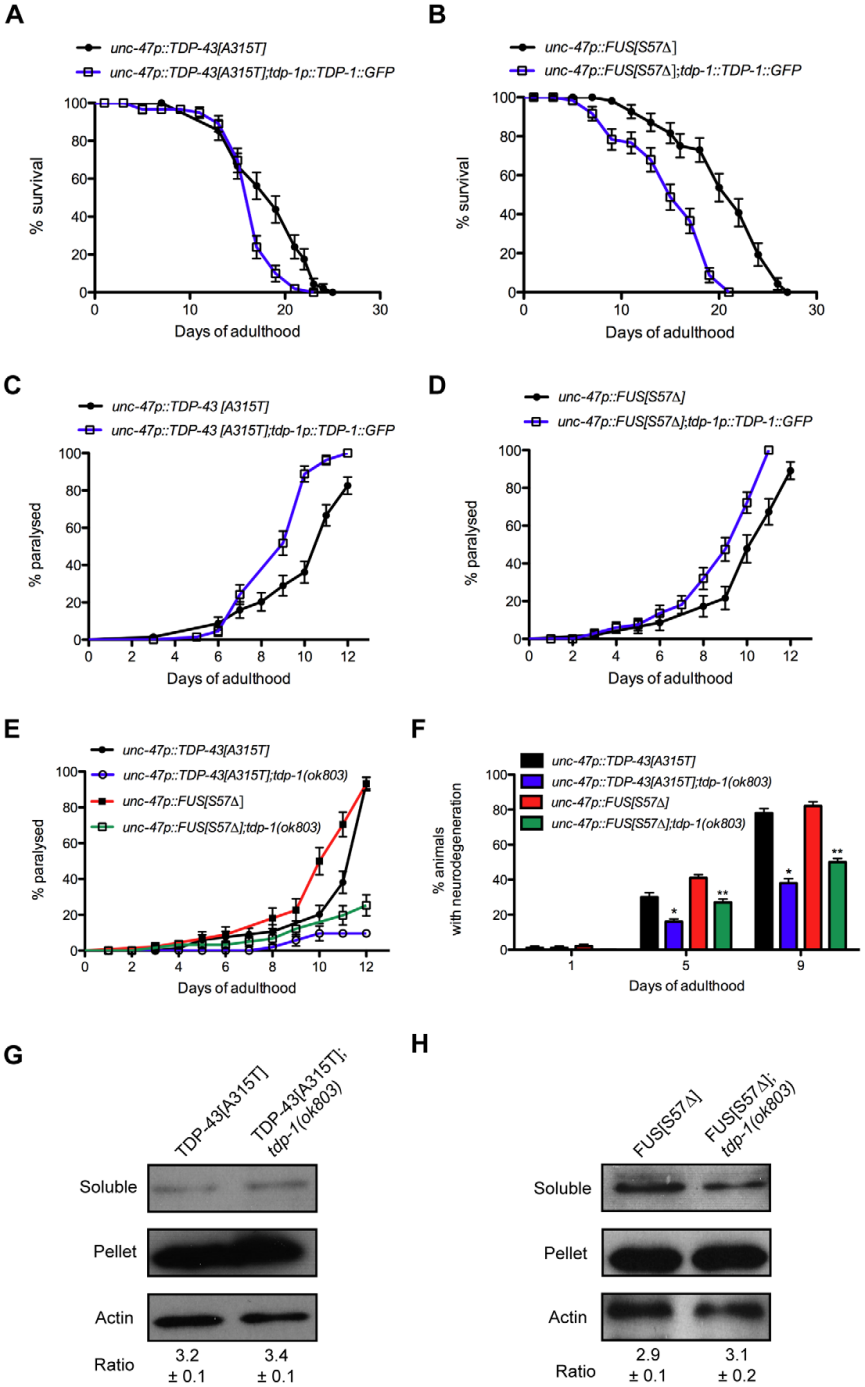
TDP-1 regulates mutant TDP-43 and FUS proteotoxicity in *C. elegans*. (A) *TDP-43[A315T];TDP-1::GFP* transgenics had shorter lifespans than transgenics expressing mutant TDP-43 alone (P<0.01). (B) *FUS[S57Δ];TDP-1::GFP* transgenics had shorter lifespans than transgenics expressing mutant FUS alone (P<0.001). Transgenics expressing (C) mutant TDP-43 or (D) mutant FUS along with TDP-1::GFP had accelerated rates of paralysis compared to transgenics expressing either mutant TDP-43 or mutant FUS alone (P<0.001 for mutant TDP-43 or FUS compared to mutant TDP-43;TDP-1::GFP or mutant FUS;TDP-1::GFP respectively). (E) Transgenics expressing mutant TDP-43 or FUS show age-dependent paralysis that is greatly reduced in worms harboring a deletion mutation of endogenous *tdp-1* (P<0.001). (F) Age-dependent motor neuron degeneration was reduced in *tdp-1(ok803);TDP-43[A315T]* and *tdp-1(ok803);FUS[S57Δ]* strains compared *to TDP-43[A315T]* or FUS[S57Δ] transgenics respectively (*, **P<0.001). Deletion of *tdp-1* did not affect the proportion of insoluble (G) mutant TDP-43 or (H) FUS proteins in extracts from whole worms. Please also see Tables S1 and S3.

Although loss of *tdp-1* sensitizes worms to oxidative and osmotic stress, elevated and chronic expression of TDP-1 leads to decreased lifespan suggesting that the induction of TDP-1 by proteotoxicity, oxidative stress or the IIS pathway may exacerbate neuronal toxicity and decrease neuronal survival. We directly tested this hypothesis by crossing TDP-1::GFP worms with our mutant TDP-43[A315T] and FUS[S57Δ] transgenics and scored for survival and the onset of paralysis. We observed that TDP-43[A315T] and FUS[S57Δ] strains containing the TDP-1::GFP transgene had significantly decreased lifespans compared to control transgenics (Figure 9A and 9B, Table S1). The expression of either mutant TDP-43[A315T] or FUS[S57Δ] causes motility defects leading to progressive paralysis and strains also expressing TDP-1::GFP had accelerated rates of paralysis compared to single transgenic controls (Figure 9C and 9D). These data suggest that induction of wild type TDP-1 expression by proteotoxicity has negative consequences on survival and neuronal function.

To rule out the possibility that the negative effects observed were simply due to transgene effects from TDP-1 overexpression we predicted that removing endogenous TDP-1 would reduce proteotoxicity. To test this we constructed *TDP-43[A315T];tdp-1(ok803)* and *FUS[S57Δ];tdp-1(ok803)* double mutant strains and observed that these animals had a significantly lower rate of paralysis compared to single transgenic TDP-43[A315T] or FUS[S57Δ] worms (Figure 9E). The expression of mutant TDP-43[A315T] or FUS[S57Δ] is accompanied by the age-dependent degeneration of motor neurons that was reduced in *tdp-1(ok803)* mutants (Figure 9F). Finally, to examine if protein misfolding was reduced in *tdp-1(ok803)* strains co-expressing mutant TDP-43 or mutant FUS, we examined the solubility of mutant TDP-43 and FUS proteins with our biochemical assay [16]. We observed no change in protein solubility after deletion of the endogenous *tdp-1* suggesting that the protective effects are not due to down-regulation or clearance of mutant proteins (Figure 9G and 9H).

## Discussion

### TDP-1 Regulates Lifespan and the Cellular Stress Response

**Figure 10:**
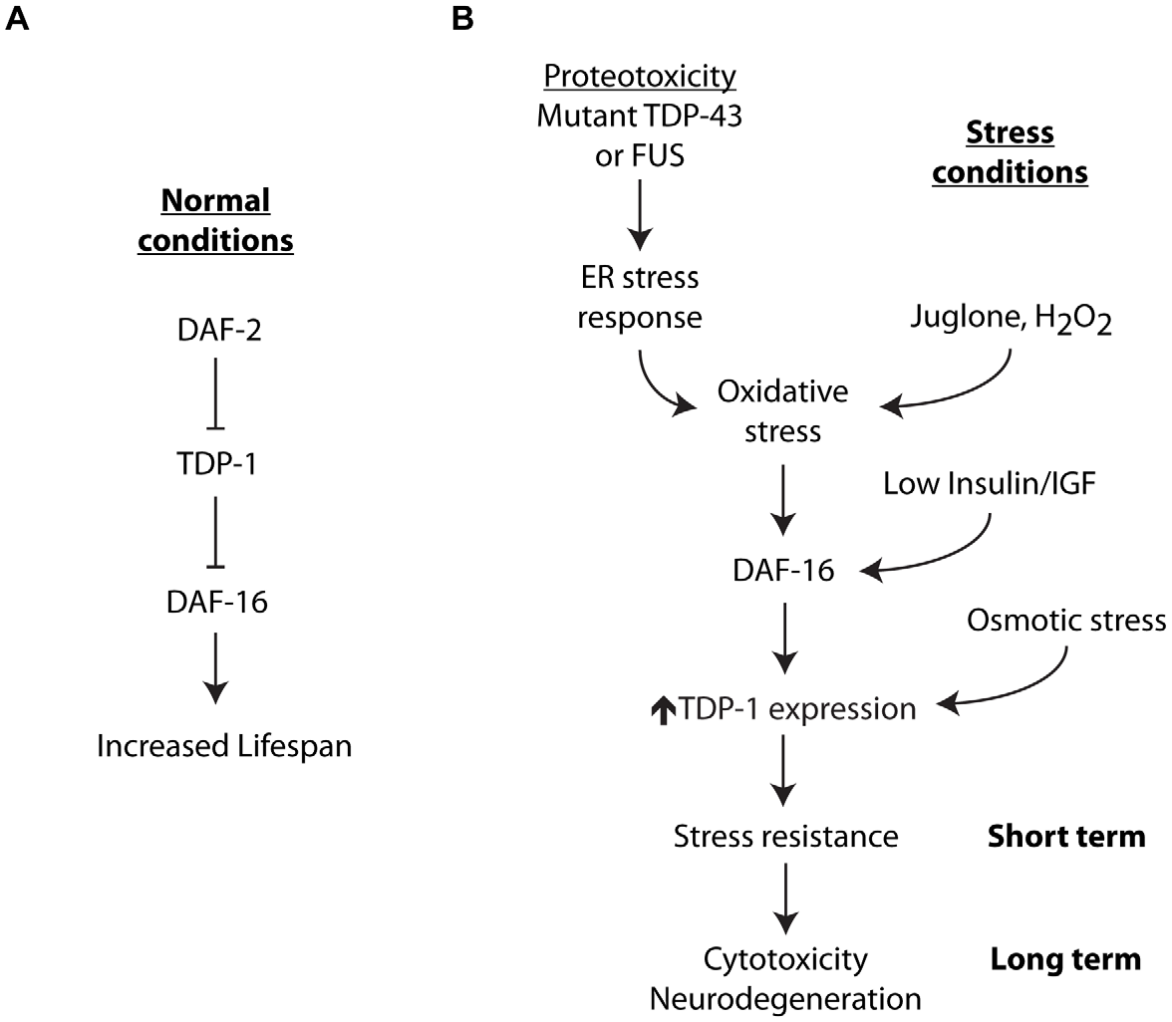
Integrated model for stress-induced TDP-1 expression. TDP-1 has multiple contributions to lifespan and the cellular stress response. (A) Under normal conditions TDP-1 may function in the Insulin/IGFP pathway upstream from DAF-16 to regulate lifespan. (B) A variety of cellular stresses induce TDP-1 expression. TDP-1 induction by oxidative stress and/or the Insulin/IGF pathway is dependent on DAF-16, while induction by osmotic stress is independent of DAF-16. Misfolded proteins activate the unfolded protein response and a secondary consequence of this is the generation of oxidative stress that in turn can induce TDP-1 expression via DAF-16. The downstream consequences of *tdp-1* expression are dependent on the length and strength of induction. *tdp-1* mutants are sensitive to stress suggesting that TDP-1 is essential for protection against acute stress. Genetically encoded proteotoxicity from proteins like mutant TDP-43 leads to chronic induction of TDP-1 expression with negative consequences including enhanced neurodegeneration. This model also suggests that mutant proteins may act as a seed for the induction of pathological TDP-1 expression.

This work identified *tdp-1* as a key stress responsive gene at the interface of longevity, stress resistance and neurodegeneration (Figure 10). The role of TDP-1 in lifespan is complex and suggests that worms like other species are sensitive to TDP-1/TDP-43 levels [23]–[26]. TDP-1 overexpression reduces lifespan while deletion of *tdp-1* in unstressed worms promotes a modest increase in lifespan but leaves worms sensitive to specific environmental stresses.

TDP-1 also has a complex role in the cellular stress response. We showed here that TDP-1 specifies the response to cellular stress with roles in oxidative, osmotic and protein-misfolding stress, but independent of high temperature, low-oxygen and damage from radiation. TDP-43 is a component of the ribonucleoprotein complexes known as stress granules that form under stressful conditions where they perform molecular mRNA triage where mRNAs are sorted for storage, degradation, or translation during stress and recovery [3]. The stress-inducible aspect of TDP-1/TDP-43 function likely reflects an ancient mechanism for enduring acute environmental stress until conditions improve. While the role of TDP-43 in response to acute stress is being actively studied [27]–[29], its role in response to chronic stresses like protein misfolding during aging is unknown.

Although the *tdp-1* alleles *ok803* and *ok781* are predicted to be molecular null alleles they did not show identical phenotypes. Both alleles extended lifespan but *ok781* did not affect several stress phenotypes. The difference between the two alleles is that *ok781* still maintains the putative Nuclear Localization Signal (Figure S1) and any potential protein product may behave differently than *ok803*. Furthermore our predictions show that the *ok781* allele may also produce an amino acid sequence with no known homology thus limiting its biological relevance. We believe the lifespan and stress phenotypes observed in the *tdp-1(ok803)* mutants are truly linked to this gene based on several lines of experimental evidence.

Concerning the lifespan phenotypes, *tdp-1*(RNAi) reduces the long-lived phenotype of *daf-2(e1370)* worms in agreement with the *tdp-1(ok803);daf-2(e1370)* strain mutant. We also tested if introducing wild type *tdp-1* DNA sequence could rescue the long-lived phenotype of *tdp-1(ok803)* mutants. Using a strain expressing a full length TDP-1 open reading frame driven by the endogenous *tdp-1* promoter we observed that it could partially rescue the extended lifespan phenotype of *tdp-1(ok803)* mutants. This is a direct proof that the lifespan phenotypes we observe in *tdp-1(ok803)* mutants is due to loss of the sequence and that we can correct this phenotype by introduction of wild type *tdp-1* sequence.

We corroborated the role of *tdp-1* in the cellular stress response with several experimental approaches. First, *tdp-1(ok803)* mutants are sensitive to oxidative and osmotic stress and we observed that our TDP-1::GFP reporter is induced by these same stresses. Second, western blotting with a TDP-1 antibody showed that endogenous TDP-1 protein levels are also induced by oxidative and osmotic stress. Third, our genetic experiments with *tdp-1(ok803)* showed that these mutants are sensitive to oxidative stress via the IIS pathway, but sensitivity to osmotic stress is independent of the IIS pathway. Our experiments with the TDP-1::GFP reporter or immunoblotting with the TDP-1 antibody fully support the observations made with the *tdp-1(ok803)* mutant. Finally, *tdp-1(ok803)* suppresses proteotoxicity while TDP-1::GFP enhances toxicity. Again, this is akin to a classic genetic rescue experiment and provides direct evidence that the phenotype observed by deletion of *tdp-1* can be modified by the introduction of wild type *tdp-1* sequence. In total we believe our hypothesis that *tdp-1* has roles in lifespan and stress is well supported by multiple approaches. It is not clear why we see very little effect for the *ok781* allele in stress assays, but at the same time there is not yet general consensus for any of the phenotypes observed for the *tdp-1* mutants.

### A Feed-Forward Model of TDP-1–Mediated Neurodegeneration

An early location for mutant TDP-43 and FUS toxicity may be within the ER. The ER has critical cellular functions including protein folding, and misfolded proteins within the ER cause stress leading to activation of the UPR^ER^ to restore homeostasis [30]. Cellular insults can lead to increased protein misfolding as can the expression of genetically encoded proteins like mutant TDP-43 or FUS [16]. Early phases of the UPR^ER^ are protective but ER stress also stimulates the clearance of misfolded proteins from the ER through ER-associated degradation (ERAD) by transporting misfolded proteins from the ER lumen to the cytoplasm for degradation by the ubiquitin-proteasome. ERAD is energetically costly, redox intense and leads to substantial production of oxidative stress and if the ER stress is not resolved it can lead to cell death [30]. We hypothesize that protein misfolding is an early step in neurodegeneration that may result in at least three overlapping mechanisms of toxicity: primary toxicity from misfolded proteins, secondary toxicity from increased oxidative stress and tertiary toxicity propagated by stress induced TDP-1 expression (Figure 10). This feed forward mechanism originating in the ER may drive cytotoxicity and neurodegeneration. If this mechanism is conserved, these data may help explain why the intracellular accumulation of wild type TDP-43 is observed in a growing number of neurodegenerative disorders. Furthermore, given TDP-43's propensity to aggregate and its inherent cytotoxicity, wild type TDP-43 may actively contribute to neurodegeneration. Indeed recent hypotheses suggest that mutant proteins may act as seeds for the accumulation of wild type TDP-43 into pathogenic conformations as described for prion toxicity [31].

Our model complements the “two-hits” hypothesis for sporadic diseases that highlights the role of environmental stresses in combination with cytoplasmic accumulations of TDP-43 as part of the trigger for pathogenesis [32]. Proteostasis is essential to survival and healthy aging but gradually becomes less efficient as organisms age and may contribute to the accumulation of TDP-43 [33]–[35]. Add to this the stress-induced expression of TDP-43 and the cell is faced with increased cytoplasmic aggregation leading to pathogenesis.

Many TDP-43 models have been described and they all show toxicity from the over expression of wild type TDP-43, which is sometimes less toxic than mutant but not always [11], [12], [21], [24], [36]–[41]. These findings again demonstrate that control of TDP-43 levels is important for cell survival and that wild type TDP-43 may contribute to neuronal toxicity. We genetically tested this premise in *C. elegans* by creating transgenic strains that either overexpressed stress-activated TDP-1 or were missing the worm's endogenous *tdp-1*. Paralysis and lifespan phenotypes of TDP-43 and FUS transgenics were worsened by increased expression of TDP-1. Consistently, deletion of endogenous *tdp-1* from strains expressing mutant TDP-43 or FUS remarkably reduced paralysis and motor neuron degeneration phenotypes. Taken together we directly showed that wild type *tdp-1* plays an important role in neurodegeneration caused by mutant TDP-43 and FUS proteins.

However two studies in *C. elegans* have shown no effect on TDP-43 toxicity in animals mutant for endogenous *tdp-1* [24], [37] while another study has shown that deleting *tdp-1* reduces TDP-43 and SOD1 toxicity [42]. The reason for the differences are not clear but may be due to differences between models, where in our model animals express mutant TDP-43 in only 26 GABAergic motor neurons [16] while the other models described rely on the expression of TDP-43 transgenes throughout the worms entire nervous system [24], [37], [42]. In our model we observe adult-onset, progressive motility defects and neurodegeneration [16], while the other models describe uncoordinated motility problems from earlier stages. It may be that the pan neuronal expression of TDP-43 in some models causes phenotypes that are too severe for modulation by reducing endogenous *tdp-1*. Additionally, the fact that we see a reduction of mutant FUS toxicity by deleting *tdp-1* bolsters the notion that proteotoxic induction of TDP-1 propagates toxicity and this may not be a phenomenon restricted to mutant TDP-43.

A surprising finding is that the UPR^ER^ appears to be activated in a cell non-autonomous manner. The *hsp-4p::GFP* reporter is expressed primarily in the worms intestinal cells while mutant TDP-43 and FUS are expressed in motor neurons. Thus ER stress generated within neurons is capable of signaling to other cells and tissue types perhaps as part of a coordinated organism wide response. Cell non-autonomous signaling has been described in *C. elegans* for mitochondrial stress regulating longevity [43] and the heat shock response [44]. Whether mutant TDP-43 and FUS similarly induce a system wide ER stress response in mammals awaits investigation.

### TDP-1 and the Insulin/IGF Pathway

Concerning the regulation of lifespan, our work describes a complex relationship between *tdp*-1 and *daf-2*. *tdp-1(ok803)* mutants have extended lifespan but are stress sensitive, while *daf-2(e1370);tdp-1(ok803)* mutants are stress sensitive and have decreased lifespan compared to *daf-2(e1370)* alone. In all systems examined increased TDP-43 expression is toxic [45] and there is widespread speculation in the field that wild type TDP-43 may contribute to cytotoxicity and neurodegeneration over long term settings [31]. Thus deleting *tdp-1* from worms leaves them sensitive to stress but frees them from potential long-term cytotoxic effects. We are not alone in the observation that removing *tdp-1* increases lifespan [42] but we are the first to look at *tdp-1*'s role in the cellular stress response. Looking at *daf-2*, the difference here may be that *tdp-1* is essential for the stress resistance aspects of *daf-2* mutants and concomitant long-lived phenotype. Thus removing *tdp-1* renders *daf-2* animals sensitive to stress induced damage limiting their extended lifespan.

A surprising finding was the opposing effects of *daf-2* mutations on proteotoxicity and TDP-1 expression. Several publications have reported that reduced *daf-2* function suppresses proteotoxicity [19]–[21]. Our experiments are not fully comparable to these studies since some studies rely solely on *daf-2*(RNAi) or on a single *daf-2* allele (*e1370*) to investigate proteotoxicity while we looked at two different *daf-2* alleles, *e1368* and *e1370*. Further complicating the matter is the fact that some models are based on muscle-cell expression vectors and/or have severe developmental effects like the recently described TDP-43 model [21].

*daf-2* mutations are grouped into a complex allelic series comprising two classes. Class 1 alleles are less severe and mutations fall within the extracellular regions of the DAF-2 receptor protein [46]. Class 2 alleles tend to be more severe, display pleiotropic effects and the mutations are found within the ligand binding area of the receptor or the tyrosine kinase domain [46]. Using the class 1 allele *daf-2(e1368)* and the class 2 allele *daf-2(e1370)* we observed that the two alleles had opposing effects on mutant TDP-43 toxicity and TDP-1 expression. In our mutant TDP-43 worms we observed that *daf-2(e1368)* suppressed mutant TDP-43 induced paralysis, while the class 2 allele *daf-2(e1370)* enhanced paralysis. Furthermore, *daf-2(e1368)* reduced the insolubility of mutant TDP-43 protein, while *daf-2(e1370)* had no effect on mutant TDP-43 solubility. Using a TDP-1::GFP reporter or western blotting with a TDP-1 antibody we observed that *daf-2(e1370)* increased the expression of TDP-1 while *daf-2(e1368)* had no effect. Given that the IIS pathway regulates both the toxicity of mutant TDP-43 proteins and the expression of endogenous TDP-1/TDP-43, altered IIS may directly contribute to ALS pathogenesis.

The pleiotropic and sometimes opposite effects of *daf-2* are known and they are consistent within each class [46]. Regarding stress, a notable example is that class 1 *daf-2(e1368)* mutants are sensitive to hypoxia while class 2 *daf-2(e1370)* mutants are highly resistant to hypoxia [47]. Here we observe opposing effects for *daf-2* on proteotoxicity and the cytotoxic induction of TDP-1 expression both of which are consistent for each allele. Our data are in agreement with studies showing that reduced IIS diminishes proteotoxicity but the question remains as to why the more severe class 2 *daf-2(e1370)* allele enhances toxicity in our TDP-43 transgenics. Part of the reason may lie with intrinsic differences between models. Our transgenics are based on the expression of TDP-43 in 26 GABAergic neurons and the worms do not show motor impairment until well into adulthood [16] while the other models show early effects and the animals are severely impaired by the time they reach adulthood [19], [21]. Thus it may be that stronger *daf-2* mutations are required to suppress severe phenotypes but are too strong for the milder, late-onset toxicity in our TDP-43 transgenics. There may be an optimal rate of IIS unique to each situation that has been referred to the Insulin Signalling Paradox [10]. Our data begin to shed light on this phenomenon where changes in signaling can have dramatically different effects and suggests that the role of IIS in neurodegeneration is more complicated that currently appreciated and requires further investigation.

### Conclusion

It is still unclear if mutant TDP-43 results in a gain, a loss of function or both but work from zebrafish and fly TDP-43 models suggest that it may be both [11], [41]. Our data introduce a novel gain of function mechanism where the increased expression of wild type TDP-1 is induced by proteotoxic stress. Several strategies come to mind to alleviate the neuronal toxicity caused by wild type and mutant TDP-43 including reducing levels of wild type TDP-43, mutant TDP-43, and/or reducing UPR^ER^ stress by promoting protein folding. In the future it will be important to determine if strategies to reduce TDP-43 neuronal toxicity may be applicable to additional neurodegenerative disease as a shared mechanism of cell death in the development of new therapeutics.

## Materials and Methods

### *C. elegans* Strains

Nematode strains used were described previously [16] or received from the *Caenorhabditis elegans* Genetics Center CGC (St Paul, MN). All strains were maintained following standard methods on OP50 bacteria plates. Strains used in this study include: *tdp-1(ok803) and tdp-1(ok781)* both outcrossed 5 times to N2 prior to use, *daf-2(e1368)*, *daf- 2(e1370)*, *daf-16(mu86)*, *gpIs1[hsp-16.2::GFP], oxIs12[unc-47p::GFP;lin-15(+)], xqIs93[tdp-1p::TDP-1::GFP], xqIs132[unc-47p::TDP-43-WT;unc-119(+)], xqIs133[unc-47p::TDP-43[A315T];unc-119(+)], xqIs173[unc-47p::FUS-WT;unc-119(+)], xqIs98[unc-47p::FUS[S57Δ];unc-119(+)], zIs356[daf-16p::daf-16-gfp; rol-6], zcIs4[hsp-4p::GFP], zcIs9[hsp-60p::GFP],gpIs1[hsp-16.2p::GFP] and zcIs13[hsp-6p::GFP].*

### Lifespan Assays

Sixty synchronized L4 were grown on OP50 bacteria plates (20 animals/plate) and three independent assays were performed. Lifespan analyses were performed at 20°C and 25°C and worms were scored every 1–2 days from adult day 1 until death. Worms were scored dead if they didn't respond to tactile or heat stimulus.

### RNAi Experiments

RNAi-treated strains were fed with *E. coli* (HT115) containing an Empty Vector (EV), *daf-16* (R13H8.1), or *tdp-1* (F44G4.4) RNAi clones from the ORFeome RNAi library (Open Biosystems). RNAi experiments were performed at 20°C. Worms were grown on NGM enriched with 1 mM Isopropyl-b-D-thiogalactopyranoside (IPTG). For lifespan, worms were transferred to RNAi 5-fluorodexyuridine (FUDR, 12.5 mg/L) plates at adult day 1 until death. Worms were declared dead if they didn't respond to tactile or heat stimulus. Experiments were conducted with 20 animals/plate by triplicates.

### Dauer Formation Assay

Young adult *daf-2(e1370)* were allowed to lay eggs overnight at 20°C. The eggs were then transferred to 25°C and scored for dauer formation 5 days later. Three different trials on different days were performed.

### Stress Assays

Stress tests were performed at 20°C (oxidative, osmotic and UV stress), 25°C (hypoxia) and 37°C (thermal stress). Worms were grown on NGM and transferred to NGM plates + 240 µM juglone (oxidative stress), or NGM plates + 10 mM Hydrogen peroxide (oxidative stress), or NGM plates + 400 mM NaCl (osmotic stress), or NGM plates + 611 mM Sorbitol (osmotic stress), all at adult day 1. For the oxidative, osmotic and thermal stress assays, worms were evaluated for survival every 30 minutes for the first 2 hours and every 2 hours after up to 14 hours; for sorbitol we also performed a test over 48 hours, starting the counts after 14 hours on the compound. For UV irradiation, adult day 1 worms were transferred to NGM plates without any food source and exposed to UV (1200 J/m2). Worms were then transferred to NGM plates with OP50 bacteria and died animals were counted every 2 hours till 14 hours after irradiation. For hypoxia experiments young adult animals were transferred to a new plate and subjected to low oxygen conditions with the AnaeroPack system (Mitsubishi Gas Chemical America) for 24 hours and assayed for survival. For all experiments nematodes were scored as dead if they were unable to move in response to heat or tactile stimuli. For all tests worms, 20 animals/plate by triplicates were scored.

### Strain Construction

Gateway system (Invitrogen) compatible *tdp-1* promoter and open reading frame plasmid clones were obtained from Open Biosystems and recombined with plasmid pDES-MB14 (kindly donated by M. Vidal, Harvard), and verified by sequencing to create a *tdp-1p::TDP-1::GFP* plasmid, which was injected at 5 ng/µl into *unc-119(ed3)* animals along with *myo-3p::mCherry*, *myo-2p::mCherry* comarkers at 5 ng/µl and wild type transformants expressing GFP were kept. The transgene was integrated using UV radiation and wild type, GFP positive animals were kept for further study. Multiple stable transgenics were isolated and outcrossed to N2 4 times before use. Strain *XQ93 xqIs93[tdp-1p::TDP-1::GFP]* was used in this study.

### Fluorescence Microscopy

For visualization of *TDP-1::GFP* animals, M9 buffer with 5 mM levamisole was used for immobilization. Animals were mounted on slides with 2% agarose pads. *TDP-1::GFP* expression was visualized with a Leica CTR 6000 and a Leica DFC 480 camera. L4 animals were grown on NGM plates and transferred to NGM plates + 240 µM juglone (oxidative stress) or NGM plates + 400 mM NaCl (osmotic stress) for 90 minutes, and examined for fluorescence with the Leica system described above. Some animals were also stained with DAPI (1∶1000, diluted in 1× PBS). Image processing was done with Adobe Photoshop. For images of TDP-1::GFP alone, images were converted to black and white and the images reversed to allow for better contrast and visualization. Quantification of TDP-1::GFP levels was done with ImageJ (NIH) and the mean and SD was calculated from 5 images for each strain and experimental condition. For visualization of *DAF-16::GFP*, *hsp-4p::GFP, hsp-6p::GFP, hsp-16.2p::GFP and hsp-60p::GFP* animals, M9 buffer with 5 mM levamisole was used for immobilization. Animals were mounted on slides with 2% agarose pads and examined for fluorescence with the Leica system described above.

### Dihydrofluorescein Diacetate Assay

For visualization of oxidative damage in the transgenic strains the worms were incubated on a slide for 30 minutes with 5 mM dihydrofluorescein diacetate dye (Sigma-Aldrich) and then washed with 1× PBS three times. After the slide was fixed fluorescence was observed with the Leica system described above.

### RT–PCR

RNA was extracted with an RNAeasy kit (Qiagen) and reverse transcribed with QuantiTect (Qiagen). Primers used include: *ctl-1* forward, AGGTCACCCATGACATCACCAAGT; *ctl-1* reverse, GAT TGCGCTTCAGGGCATGAATGA; *ctl-2* forward, TTCGCTGAGTTGAACAATCCG; *ctl-2* reverse, GTTGCTGATTGTCATAAGCCATTGC; *tdp-1* forward, AAAGTGGGATCGAGTGACGAC; *tdp-1* reverse, GACAGCGTAACGAATGCAAAGC; *sod-3* forward, CGAGCTCGAACCTGTAATCAGCCATG; *sod-3* reverse, GGGGTACCGCTGATATTCTTCCACTTG; *act-3* forward, GTTGCCGCTCTTGTTGTAGAC; *act-3* reverse, GGAGAGGACAGCTTGGATGG.

### Worm Lysates

Worms were collected in M9 buffer, washed 3 times with M9 and pellets were placed at −80°C overnight. Pellets were lysed in RIPA buffer (150 mM NaCl, 50 mM Tris pH 7.4, 1% Triton X-100, 0.1% SDS, 1% sodium deoxycholate) + 0.1% protease inhibitors (10 mg/ml leupeptin, 10 mg/ml pepstatin A, 10 mg/ml chymostatin LPC;1/1000). Pellets were passed through a 27_1/2_ G syringe 10 times, sonicated and centrifuged at 16000 *g*. Supernatants were collected.

### Protein Solubility

For TDP-43 and FUS transgenics, soluble/insoluble fractions, worms were lysed in Extraction Buffer (1 M Tris-HCl pH 8, 0.5 M EDTA, 1 M NaCl, 10% NP40 + protease inhibitors (LPC;1/1000). Pellets were passed through a 27_1/2_ G syringe 10 times, sonicated and centrifuged at 100000 *g* for 5 minutes. The soluble supernatant was saved and the remaining pellet was resuspended in extraction buffer, sonicated and centrifuged at 100000 g for 5 minutes. The remaining pellet was resuspended into 100 µl of RIPA buffer, sonicated until the pellet was resuspended in solution and saved.

### Protein Quantification

All supernatants were quantified with the BCA Protein Assay Kit (Thermo Scientific) following the manufacturer instructions.

### Immunoblot

Worm RIPA samples (175 µg/well for transgenic worms; 15 µg/well for non transgenics) were resuspended directly in 1× Laemmli sample buffer, migrated in 10% polyacrylamide gels, transferred to nitrocellulose membranes (BioRad) and immunoblotted. Antibodies used: rabbit anti-TDP-43 (1∶200, Proteintech), rabbit anti-FUS/TLS (1∶200, Abcam), rabbit anti-TDP-1 (1∶500, Petrucelli laboratory) and mouse anti-Actin (1∶10000, MP Biomedical). Blots were visualized with peroxidase-conjugated secondary antibodies and ECL Western Blotting Substrate (Thermo Scientific). Densitometry was performed with Photoshop (Adobe).

### Statistics

For lifespan and stress-resistance tests, survival curves were generated and compared using the Log-rank (Mantel-Cox) test, and 20–30 animals were tested per genotype and repeated at least three times. For progeny counts, dauer-formation assays and hypoxia tests the mean and SEM were calculated for each trial and two-tailed *t*-tests were used for statistical analysis.

## Supporting Information

Figure S1 Human TDP-43 and *C. elegans* TDP-1. A) Comparison of human TDP-43 and *C. elegans* TDP-1 proteins. The extent of the deletion allele *ok803* is indicated. RRM: RNA Recognition Motif, NLS: Nuclear Localization Signal. (B) Detection of the deletion alleles *tdp-1(ok781)* and *tdp-1(ok803)* by PCR with oligonucleotides spanning both deletions. Non-mutant N2 worms produce a band of ∼1600 bp, *tdp-1(ok803)* an ∼430 bp band, and *tdp-1(ok781)* a ∼480 bp band.
(TIF)

Figure S2 *tdp-1* is not required for dauer formation or resistance to heat, hypoxia or radiation. (A) *tdp-1(ok803)* did not interfere with the constitutive dauer-formation phenotype of *daf-2(e1370)* animals grown at 25°C. (B) *tdp-1(ok803)* worms were more sensitive to osmotic stress from sorbitol than wild type N2 worms (P<0.001). *daf-2(e1370)* and *daf-2(e1370);tdp-1(ok803)* were statistically indistinguishable from one another in their response to sorbitol but were both highly resistant compared to N2 controls (P<0.001). (C) *tdp-1(ok803)* mutants and N2 worms showed similar sensitivity to thermal stress. *daf-2(e1370)* and *daf-2(e1370);tdp-1(ok803)* mutants were highly resistant to thermal stress compared to N2 controls (P<0.001). (D) Wild type N2 worms and *tdp-1(ok803)* mutants were both highly susceptible to mortality caused by low oxygen conditions. *tdp-1(ok803)* did not interfere with the resistance of *daf-2(e1370)* animals against hypoxia. (E) *tdp-1(ok803)* mutants and N2 worms were equally sensitive to UV radiation. *daf-2(e1370)* and *daf-2(e1370);tdp-1(ok803)* mutants were both highly resistant to UV compared to N2 controls (P<0.001).
(TIF)

Figure S3 *tdp-1(ok781)* has variable phenotypes. (A) *tdp-1(ok781)* mutants show a modest but significant increase in lifespan compared to N2 worms. *tdp-1(ok781)* worms were indistinguishable from N2 worms in their response to (B) juglone induced oxidative stress, (C) NaCl induced osmotic stress and (D) thermal stress. Please also see Table S4.
(TIF)

Figure S4 Quantification of TDP-1::GFP expression. (A) Quantification of TDP-1::GFP expression in transgenics exposed to osmotic stress (NaCl) or juglone oxidative stress (juglone). *P<0.001 compared to untreated animals. Linked to Figure 3E. (B) Quantification of fluorescence in *tdp-1(ok803)*;TDP-1::GFP in untreated and animals exposed to either NaCl or juglone. *P<0.001 compared to untreated animals. Linked to Figure 3F. (C) Measurement of fluorescence in *daf-2(e1370)*;TDP-1::GFP and *daf-2(e1368)*;TDP-1::GFP animals under normal or stressed conditions. *P<0.001 compared to untreated animals. Linked to Figure 3G and 3F. (D) Measurement of fluorescence in *daf-16(mu86)*;TDP-1::GFP animals under normal and stress conditions. *P<0.001 compared to untreated animals. Linked to Figure 3I. (E) Measurement of fluorescence in *daf-2(e1370)*;TDP-1::GFP animals exposed to *daf-16*(RNAi) or empty vector controls. *P<0.001 compared to untreated animals. Linked to Figure S5A and S5B. (F) Measurement of fluorescence from the TDP-1::GFP transgene in various TDP-43 and FUS transgenics. Tun: Tunicamycin. *P<0.001 compared to untreated animals. Linked to Figure 6.
(TIF)

Figure S5 Stress assays do not induce general expression of transgenes. (A) Oxidative or osmotic stress did not induce the expression of *unc-47p::GFP* compared to untreated controls. (B) *sod-3p::GFP* expression was induced by oxidative or osmotic stress compared to untreated controls.
(TIF)

Figure S6 TDP-1 functions downstream of DAF-16. (A) TDP-1::GFP expression was upregulated in *daf-2(e1370)* mutants and was unaffected by empty vector RNAi controls. (B) *daf-16*(RNAi) abolished the increased expression of TDP-1::GFP in *daf-2(e1370)* mutants. (C) DAF-16::GFP is cytoplasmic and diffuse under normal conditions but is (D) localized to nuclei when the animals are exposed to juglone. (E) The GFP signal is diffuse in unstressed *daf-16::GFP;tdp-1(ok803)* transgenics and (E) localizes within the nuclei of transgenics treated with juglone.
(TIF)

Figure S7 Characterization of the TDP-1 antibody. Western blotting of protein extracts from wild type N2, *tdp-1(ok803)* and *tdp-1(ok781)* strains with a polyclonal anti TDP-1 antibody revealed a signal in N2 worms but no signals from the deletion mutants *ok803* or *ok781*.
(TIF)

Figure S8 Mutant TDP-43 does not activate mitochondrial or cytoplasmic chaperones. (A–I) are low-resolution photographs of young adult transgenic worms. The images have been converted to black and white and photo-reversed to aid visualization. Difficult to see worms are outlined. (A) Low expression of the mitochondrial chaperone reporter *hsp-6p::GFP* under non-stress conditions. (B) Ethidium bromide induced *hsp-6p::GFP* expression. (C) Mutant TDP-43 did not induce *hsp-6p::GFP* expression. (D) Low expression of the mitochondrial chaperone reporter *hsp-60p::GFP* under non-stress conditions. (E) Ethidium bromide induced *hsp-60p::GFP* expression. (F) Mutant TDP-43 did not induce *hsp-60p::GFP* expression. (G) Low expression of the heat shock reporter *hsp-16.2p::GFP* under non-stress conditions. (H) High temperatures induced *hsp-16.2p::GFP* expression. (I) Mutant TDP-43 did not induce *hsp-16.2p::GFP* expression.
(TIF)

Table S1 Lifespan analysis for all experiments. Related to Figure 1 and Figure 9. Animals that died prematurely (ruptured, internal hatching) or were lost (crawled off the plate) were censored at the time of scoring. All control and experimental animals were scored and transferred to new plates at the same time. n.s. not significant.
(PDF)

Table S2 Stress assays. Related to Figure 2 and Figure S2. Animals were examined every two hours for survival against the specified stress.
(PDF)

Table S3 Paralysis tests. Related to Figure 8 and Figure 9.
(PDF)

Table S4 Lifespan and stress assays for *tdp-1(ok781)*. Related to Figure S3. Animals that died prematurely (ruptured, internal hatching) or were lost (crawled off the plate) were censored at the time of scoring. All control and experimental animals were scored and transferred to new plates at the same time. For the stress assays, animals were examined every two hours for survival against the specified stress. n.s. not significant.
(PDF)

## References

[1] Lagier-Tourenne C, Polymenidou M, Cleveland DW (2010). TDP-43 and FUS/TLS: emerging roles in RNA processing and neurodegeneration.. Hum Mol Genet.

[2] Anderson P, Kedersha N (2009). Stress granules.. Curr Biol.

[3] Anderson P, Kedersha N (2009). RNA granules: post-transcriptional and epigenetic modulators of gene expression.. Nat Rev Mol Cell Biol.

[4] Kenyon C (2005). The plasticity of aging: insights from long-lived mutants.. Cell.

[5] Kenyon C, Chang J, Gensch E, Rudner A, Tabtiang R (1993). A C. elegans mutant that lives twice as long as wild type.. Nature.

[6] Libina N, Berman JR, Kenyon C (2003). Tissue-specific activities of C. elegans DAF-16 in the regulation of lifespan.. Cell.

[7] Lin K, Dorman JB, Rodan A, Kenyon C (1997). daf-16: An HNF-3/forkhead family member that can function to double the life-span of Caenorhabditis elegans.. Science.

[8] Lin K, Hsin H, Libina N, Kenyon C (2001). Regulation of the Caenorhabditis elegans longevity protein DAF-16 by insulin/IGF-1 and germline signaling.. Nat Genet.

[9] Murphy CT, McCarroll SA, Bargmann CI, Fraser A, Kamath RS (2003). Genes that act downstream of DAF-16 to influence the lifespan of Caenorhabditis elegans.. Nature.

[10] Cohen E, Dillin A (2008). The insulin paradox: aging, proteotoxicity and neurodegeneration.. Nat Rev Neurosci.

[11] Estes PS, Boehringer A, Zwick R, Tang JE, Grigsby B (2011). Wild-type and A315T mutant TDP-43 exert differential neurotoxicity in a Drosophila model of ALS.. Hum Mol Genet.

[12] Xu YF, Gendron TF, Zhang YJ, Lin WL, D'Alton S (2010). Wild-type human TDP-43 expression causes TDP-43 phosphorylation, mitochondrial aggregation, motor deficits, and early mortality in transgenic mice.. J Neurosci.

[13] Van Raamsdonk JM, Hekimi S (2009). Deletion of the mitochondrial superoxide dismutase sod-2 extends lifespan in Caenorhabditis elegans.. PLoS Genet.

[14] Henderson ST, Johnson TE (2001). daf-16 integrates developmental and environmental inputs to mediate aging in the nematode Caenorhabditis elegans.. Curr Biol.

[15] Douglas PM, Dillin A (2010). Protein homeostasis and aging in neurodegeneration.. J Cell Biol.

[16] Vaccaro A, Tauffenberger A, Aggad D, Rouleau G, Drapeau P (2012). Mutant TDP-43 and FUS Cause Age-Dependent Paralysis and Neurodegeneration in C. elegans.. PLoS ONE.

[17] Urano F, Calfon M, Yoneda T, Yun C, Kiraly M (2002). A survival pathway for Caenorhabditis elegans with a blocked unfolded protein response.. J Cell Biol.

[18] Harding HP, Zhang Y, Zeng H, Novoa I, Lu PD (2003). An integrated stress response regulates amino acid metabolism and resistance to oxidative stress.. Mol Cell.

[19] Cohen E, Bieschke J, Perciavalle RM, Kelly JW, Dillin A (2006). Opposing activities protect against age-onset proteotoxicity.. Science.

[20] Teixeira-Castro A, Ailion M, Jalles A, Brignull HR, Vilaca JL (2011). Neuron-specific proteotoxicity of mutant ataxin-3 in C. elegans: rescue by the DAF-16 and HSF-1 pathways.. Hum Mol Genet.

[21] Zhang T, Mullane PC, Periz G, Wang J (2011). TDP-43 neurotoxicity and protein aggregation modulated by heat shock factor and insulin/IGF-1 signaling.. Hum Mol Genet.

[22] Parker JA, Arango M, Abderrahmane S, Lambert E, Tourette C (2005). Resveratrol rescues mutant polyglutamine cytotoxicity in nematode and mammalian neurons.. Nat Genet.

[23] Ayala YM, De Conti L, Avendano-Vazquez SE, Dhir A, Romano M (2011). TDP-43 regulates its mRNA levels through a negative feedback loop.. EMBO J.

[24] Ash PE, Zhang YJ, Roberts CM, Saldi T, Hutter H (2010). Neurotoxic effects of TDP-43 overexpression in C. elegans.. Hum Mol Genet.

[25] Wegorzewska I, Bell S, Cairns NJ, Miller TM, Baloh RH (2009). TDP-43 mutant transgenic mice develop features of ALS and frontotemporal lobar degeneration.. Proc Natl Acad Sci U S A.

[26] Johnson BS, McCaffery JM, Lindquist S, Gitler AD (2008). A yeast TDP-43 proteinopathy model: Exploring the molecular determinants of TDP-43 aggregation and cellular toxicity.. Proc Natl Acad Sci U S A.

[27] Colombrita C, Zennaro E, Fallini C, Weber M, Sommacal A (2009). TDP-43 is recruited to stress granules in conditions of oxidative insult.. J Neurochem.

[28] Bosco DA, Lemay N, Ko HK, Zhou H, Burke C (2010). Mutant FUS Proteins that Cause Amyotrophic Lateral Sclerosis Incorporate into Stress Granules.. Hum Mol Genet.

[29] McDonald KK, Aulas A, Destroismaisons L, Pickles S, Beleac E (2011). TAR DNA-binding protein 43 (TDP-43) regulates stress granule dynamics via differential regulation of G3BP and TIA-1.. Hum Mol Genet.

[30] Walker AK, Atkin JD (2011). Stress signaling from the endoplasmic reticulum: A central player in the pathogenesis of amyotrophic lateral sclerosis.. IUBMB Life.

[31] Polymenidou M, Cleveland DW (2011). The Seeds of Neurodegeneration: Prion-like Spreading in ALS.. Cell.

[32] Pesiridis GS, Tripathy K, Tanik S, Trojanowski JQ, Lee VM (2011). A “two-hit” hypothesis for inclusion formation by carboxyl-terminal fragments of TDP-43 protein linked to RNA depletion and impaired microtubule-dependent transport.. J Biol Chem.

[33] Makrides SC (1983). Protein synthesis and degradation during aging and senescence.. Biol Rev Camb Philos Soc.

[34] Partridge L, Gems D (2002). Mechanisms of ageing: public or private?. Nat Rev Genet.

[35] Rattan SI, Clark BF (1996). Intracellular protein synthesis, modifications and aging.. Biochem Soc Trans.

[36] Wils H, Kleinberger G, Janssens J, Pereson S, Joris G (2010). TDP-43 transgenic mice develop spastic paralysis and neuronal inclusions characteristic of ALS and frontotemporal lobar degeneration.. Proc Natl Acad Sci U S A.

[37] Liachko NF, Guthrie CR, Kraemer BC (2010). Phosphorylation Promotes Neurotoxicity in a Caenorhabditis elegans Model of TDP-43 Proteinopathy.. J Neurosci.

[38] Li Y, Ray P, Rao EJ, Shi C, Guo W (2010). A Drosophila model for TDP-43 proteinopathy.. Proc Natl Acad Sci U S A.

[39] Barmada SJ, Skibinski G, Korb E, Rao EJ, Wu JY (2010). Cytoplasmic mislocalization of TDP-43 is toxic to neurons and enhanced by a mutation associated with familial amyotrophic lateral sclerosis.. J Neurosci.

[40] Zhang YJ, Xu YF, Cook C, Gendron TF, Roettges P (2009). Aberrant cleavage of TDP-43 enhances aggregation and cellular toxicity.. Proc Natl Acad Sci U S A.

[41] Kabashi E, Lin L, Tradewell ML, Dion PA, Bercier V (2009). Gain and loss of function of ALS-related mutations of TARDBP (TDP-43) cause motor deficits in vivo.. Hum Mol Genet.

[42] Zhang T, Hwang HY, Hao H, Talbot C, Wang J (2012). Caenorhabditis elegans RNA-processing Protein TDP-1 Regulates Protein Homeostasis and Lifespan.. J Biol Chem.

[43] Durieux J, Wolff S, Dillin A (2011). The cell-non-autonomous nature of electron transport chain-mediated longevity.. Cell.

[44] Prahlad V, Cornelius T, Morimoto RI (2008). Regulation of the cellular heat shock response in Caenorhabditis elegans by thermosensory neurons.. Science.

[45] Da Cruz S, Cleveland DW (2011). Understanding the role of TDP-43 and FUS/TLS in ALS and beyond.. Curr Opin Neurobiol.

[46] Patel DS, Garza-Garcia A, Nanji M, McElwee JJ, Ackerman D (2008). Clustering of genetically defined allele classes in the Caenorhabditis elegans DAF-2 insulin/IGF-1 receptor.. Genetics.

[47] Scott BA, Avidan MS, Crowder CM (2002). Regulation of hypoxic death in C. elegans by the insulin/IGF receptor homolog DAF-2.. Science.

