# Supplementary figures and images for: TDP-1/TDP-43 Regulates Stress Signaling and Age-Dependent Proteotoxicity in Caenorhabditis elegans
Source: PLoS Genet. 2012 Jul 5;8(7):e1002806. doi: 10.1371/journal.pgen.1002806 (PMC3390363; doi:10.1371/journal.pgen.1002806)

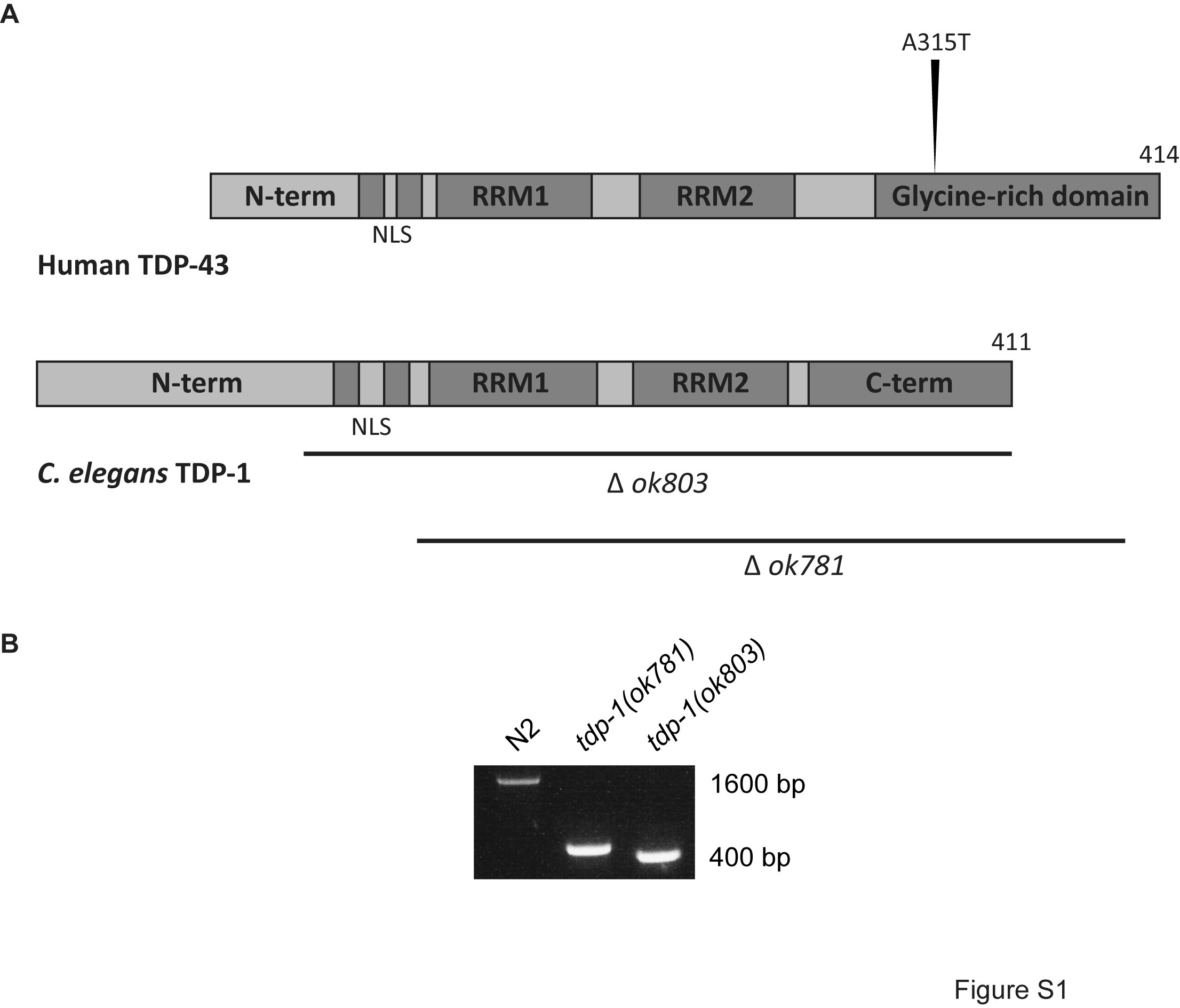

Supplement: Figure S1 — Human TDP-43 and C. elegans TDP-1. A) Comparison of human TDP-43 and C. elegans TDP-1 proteins. The extent of the deletion allele ok803 is indicated. RRM: RNA Recognition Motif, NLS: Nuclear Localization Signal. (B) Detection of the deletion alleles tdp-1(ok781) and tdp-1(ok803) by PCR with oligonucleotides spanning both deletions. Non-mutant N2 worms produce a band of ∼1600 bp, tdp-1(ok803) an ∼430 bp band, and tdp-1(ok781) a ∼480 bp band. (TIF) [file pgen.1002806.s001.tif]

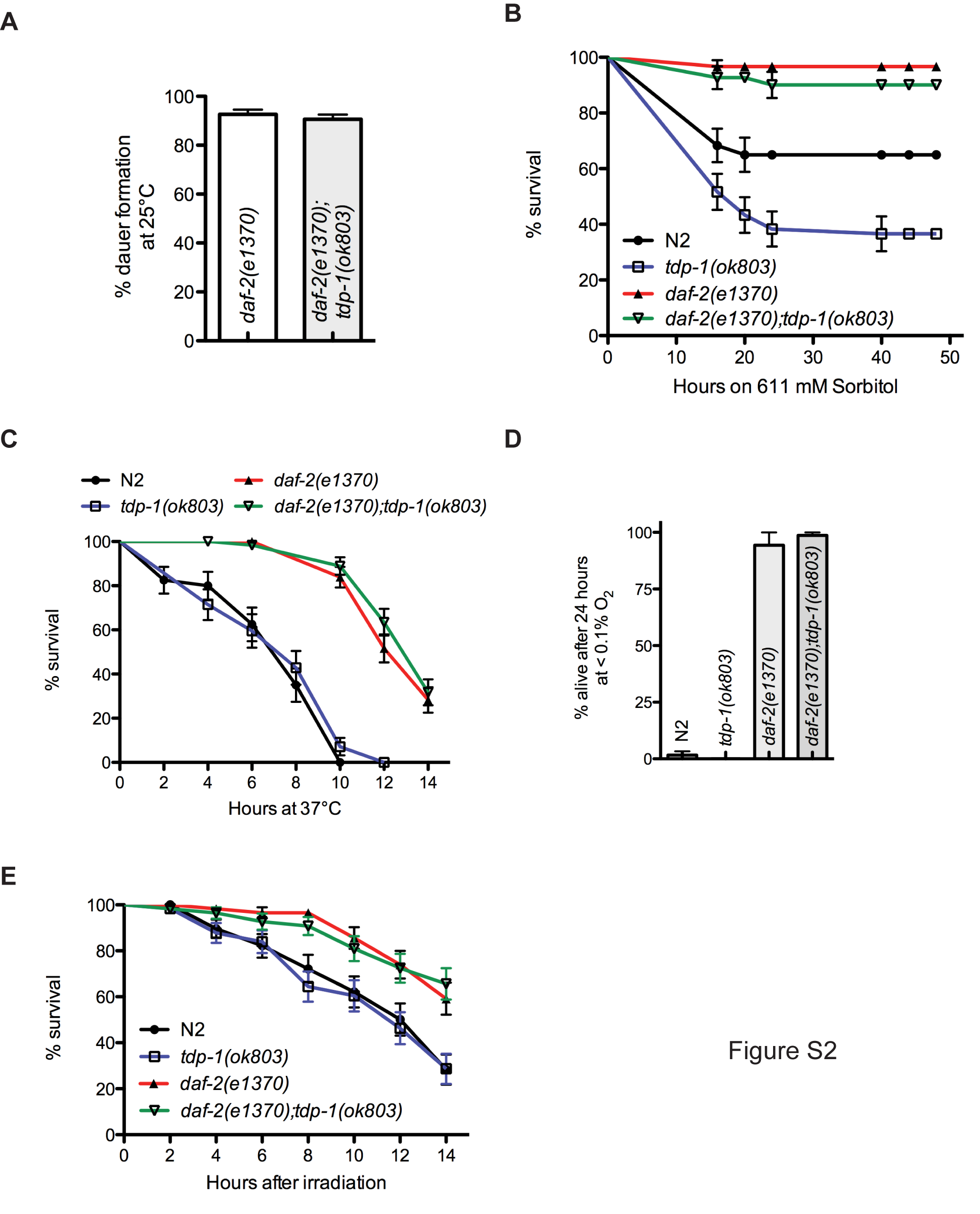

Supplement: Figure S2 — tdp-1 is not required for dauer formation or resistance to heat, hypoxia or radiation. (A) tdp-1(ok803) did not interfere with the constitutive dauer-formation phenotype of daf-2(e1370) animals grown at 25°C. (B) tdp-1(ok803) worms were more sensitive to osmotic stress from sorbitol than wild type N2 worms (P<0.001). daf-2(e1370) and daf-2(e1370);tdp-1(ok803) were statistically indistinguishable from one another in their response to sorbitol but were both highly resistant compared to N2 controls (P<0.001). (C) tdp-1(ok803) mutants and N2 worms showed similar sensitivity to thermal stress. daf-2(e1370) and daf-2(e1370);tdp-1(ok803) mutants were highly resistant to thermal stress compared to N2 controls (P<0.001). (D) Wild type N2 worms and tdp-1(ok803) mutants were both highly susceptible to mortality caused by low oxygen conditions. tdp-1(ok803) did not interfere with the resistance of daf-2(e1370) animals against hypoxia. (E) tdp-1(ok803) mutants and N2 worms were equally sensitive to UV radiation. daf-2(e1370) and daf-2(e1370);tdp-1(ok803) mutants were both highly resistant to UV compared to N2 controls (P<0.001). (TIF) [file pgen.1002806.s002.tif]

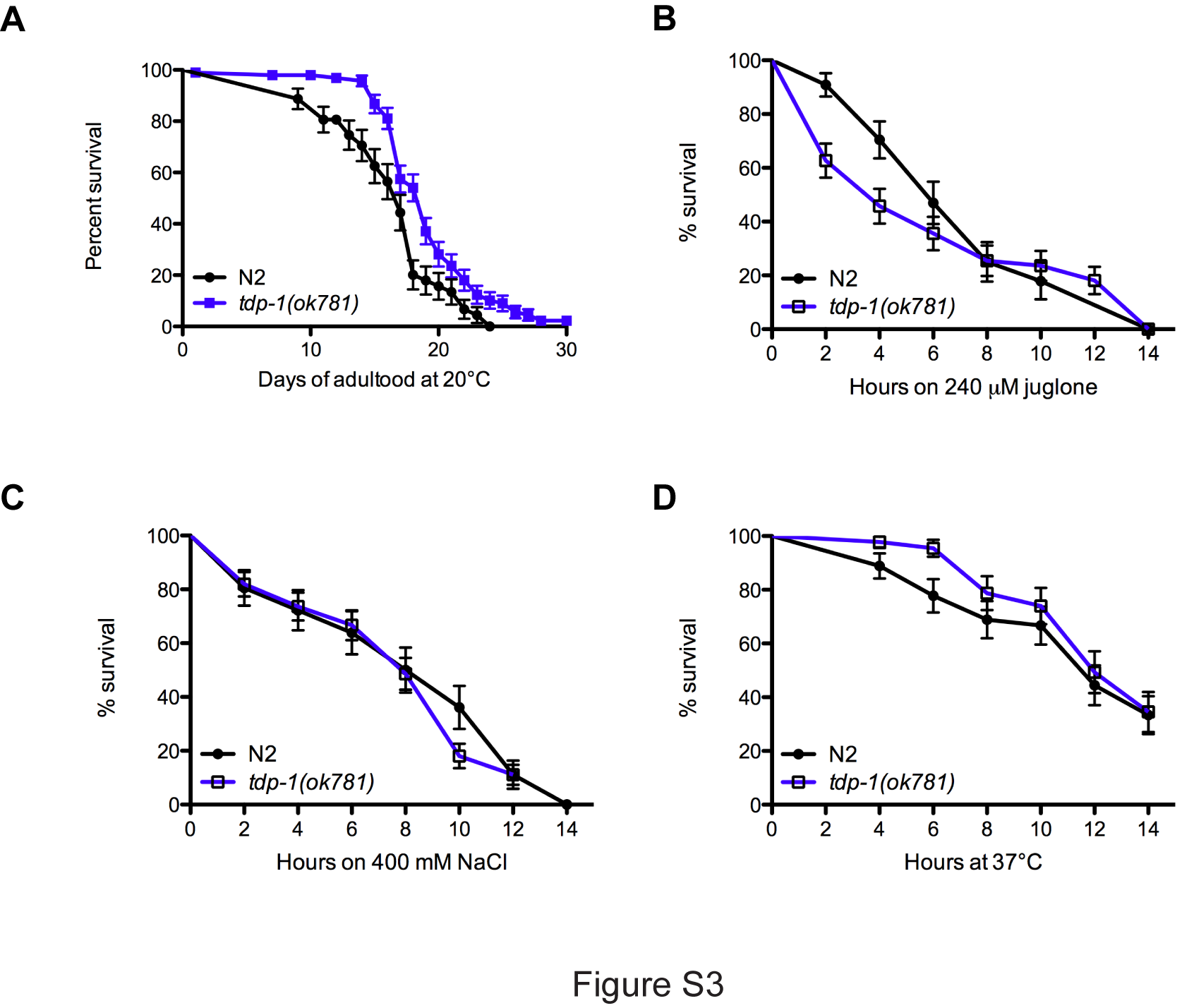

Supplement: Figure S3 — tdp-1(ok781) has variable phenotypes. (A) tdp-1(ok781) mutants show a modest but significant increase in lifespan compared to N2 worms. tdp-1(ok781) worms were indistinguishable from N2 worms in their response to (B) juglone induced oxidative stress, (C) NaCl induced osmotic stress and (D) thermal stress. Please also see Table S4. (TIF) [file pgen.1002806.s003.tif]

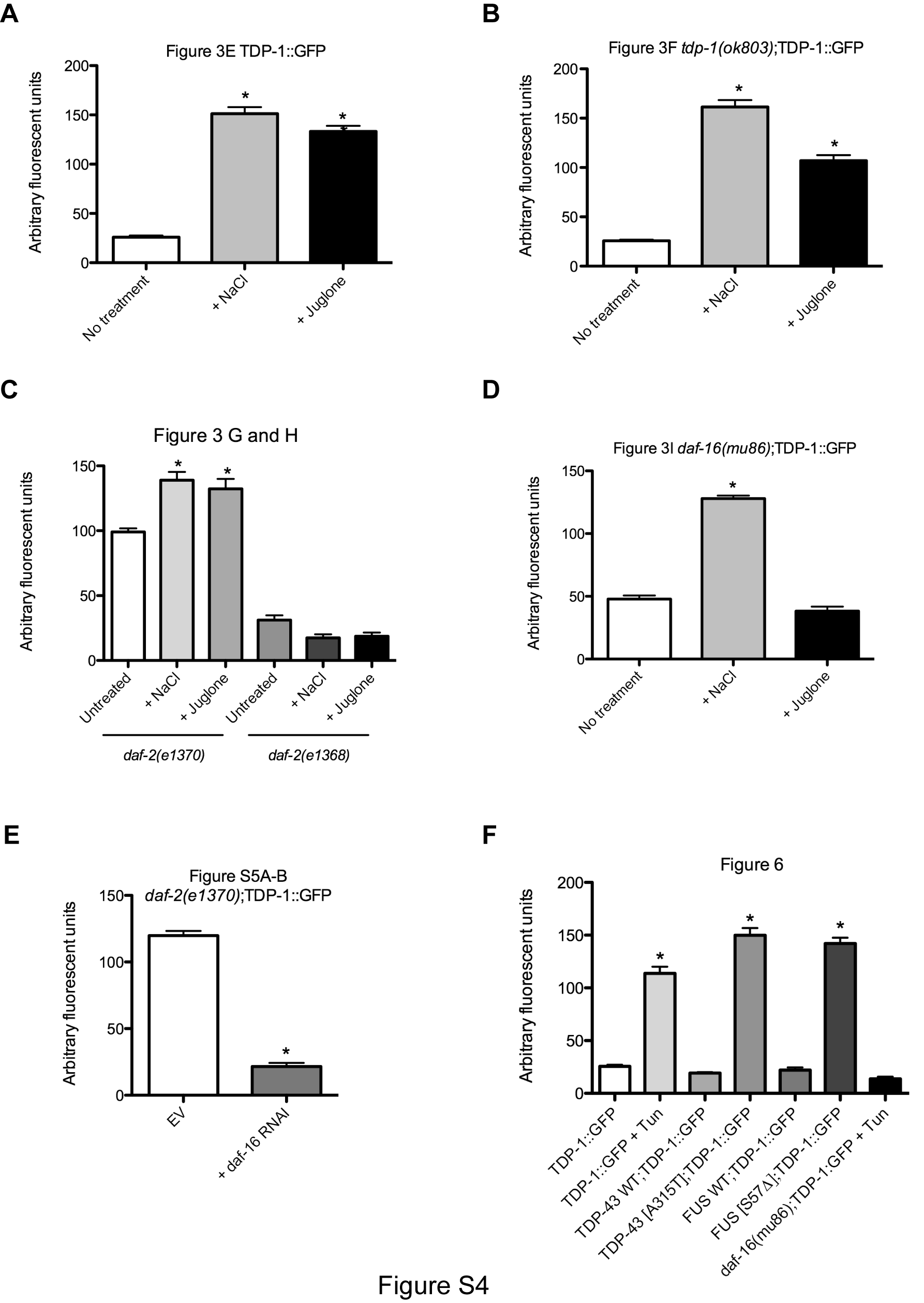

Supplement: Figure S4 — Quantification of TDP-1::GFP expression. (A) Quantification of TDP-1::GFP expression in transgenics exposed to osmotic stress (NaCl) or juglone oxidative stress (juglone). *P<0.001 compared to untreated animals. Linked to Figure 3E. (B) Quantification of fluorescence in tdp-1(ok803);TDP-1::GFP in untreated and animals exposed to either NaCl or juglone. *P<0.001 compared to untreated animals. Linked to Figure 3F. (C) Measurement of fluorescence in daf-2(e1370);TDP-1::GFP and daf-2(e1368);TDP-1::GFP animals under normal or stressed conditions. *P<0.001 compared to untreated animals. Linked to Figure 3G and 3F. (D) Measurement of fluorescence in daf-16(mu86);TDP-1::GFP animals under normal and stress conditions. *P<0.001 compared to untreated animals. Linked to Figure 3I. (E) Measurement of fluorescence in daf-2(e1370);TDP-1::GFP animals exposed to daf-16(RNAi) or empty vector controls. *P<0.001 compared to untreated animals. Linked to Figure S5A and S5B. (F) Measurement of fluorescence from the TDP-1::GFP transgene in various TDP-43 and FUS transgenics. Tun: Tunicamycin. *P<0.001 compared to untreated animals. Linked to Figure 6. (TIF) [file pgen.1002806.s004.tif]

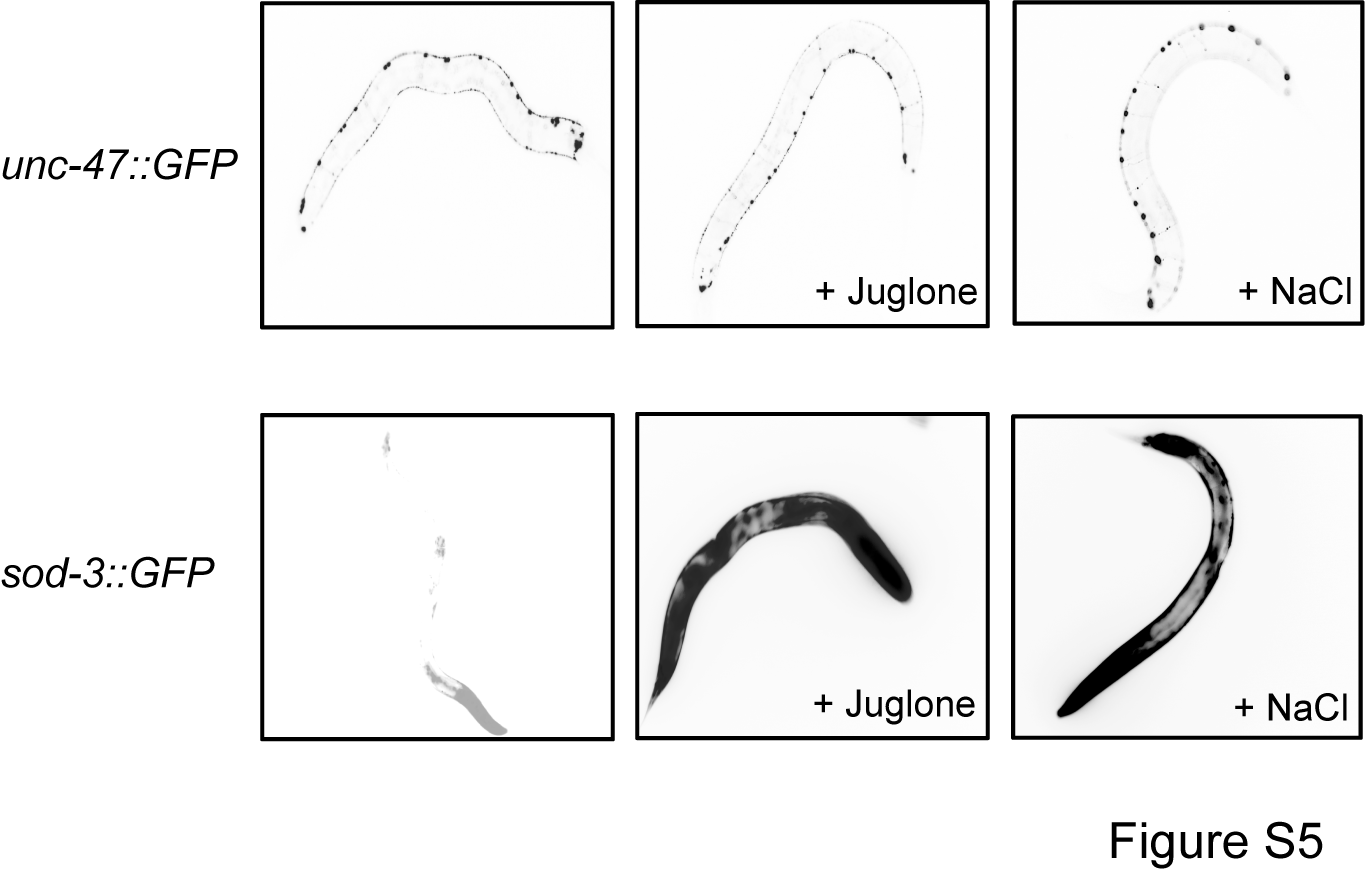

Supplement: Figure S5 — Stress assays do not induce general expression of transgenes. (A) Oxidative or osmotic stress did not induce the expression of unc-47p::GFP compared to untreated controls. (B) sod-3p::GFP expression was induced by oxidative or osmotic stress compared to untreated controls. (TIF) [file pgen.1002806.s005.tif]

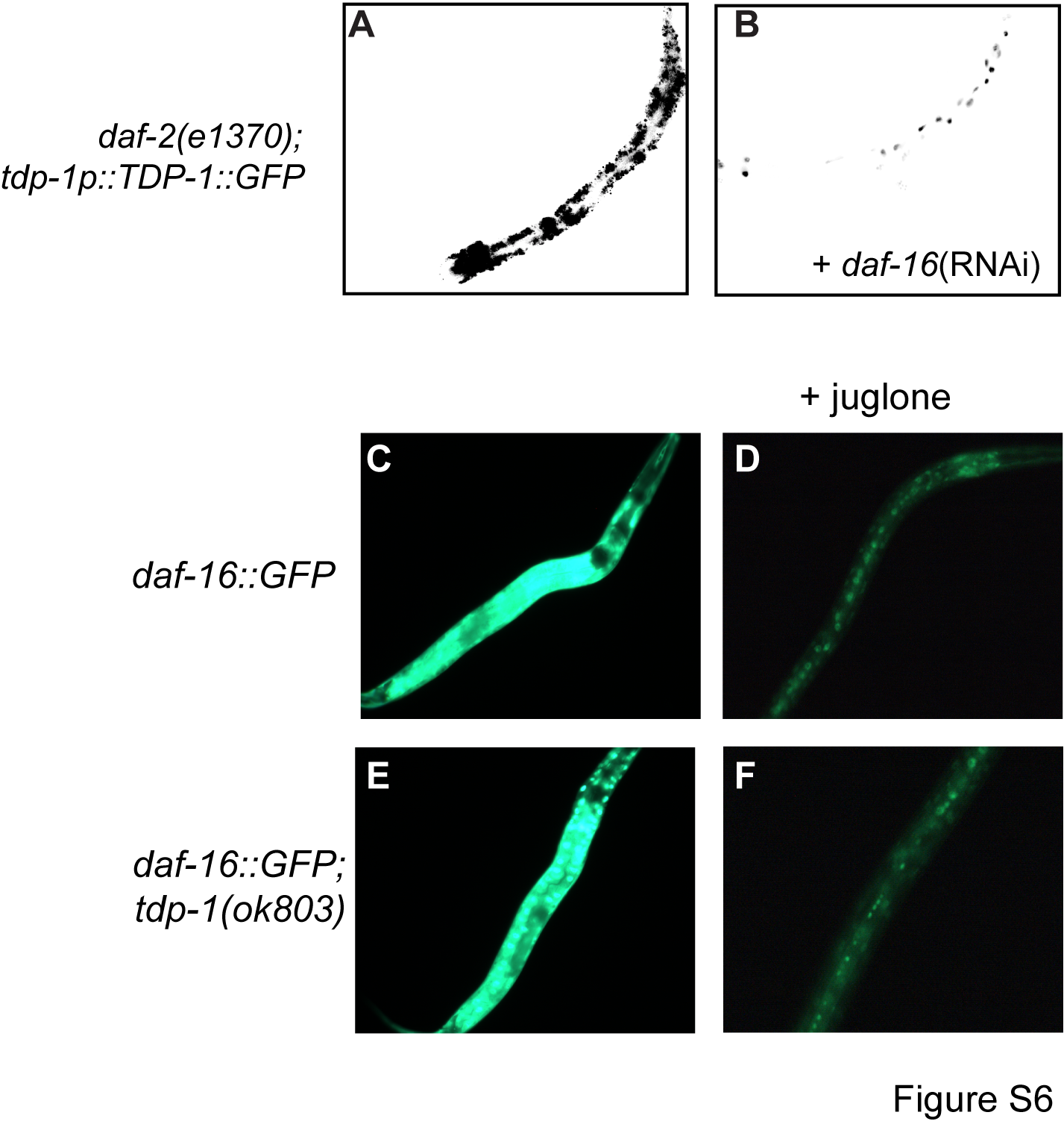

Supplement: Figure S6 — TDP-1 functions downstream of DAF-16. (A) TDP-1::GFP expression was upregulated in daf-2(e1370) mutants and was unaffected by empty vector RNAi controls. (B) daf-16(RNAi) abolished the increased expression of TDP-1::GFP in daf-2(e1370) mutants. (C) DAF-16::GFP is cytoplasmic and diffuse under normal conditions but is (D) localized to nuclei when the animals are exposed to juglone. (E) The GFP signal is diffuse in unstressed daf-16::GFP;tdp-1(ok803) transgenics and (E) localizes within the nuclei of transgenics treated with juglone. (TIF) [file pgen.1002806.s006.tif]

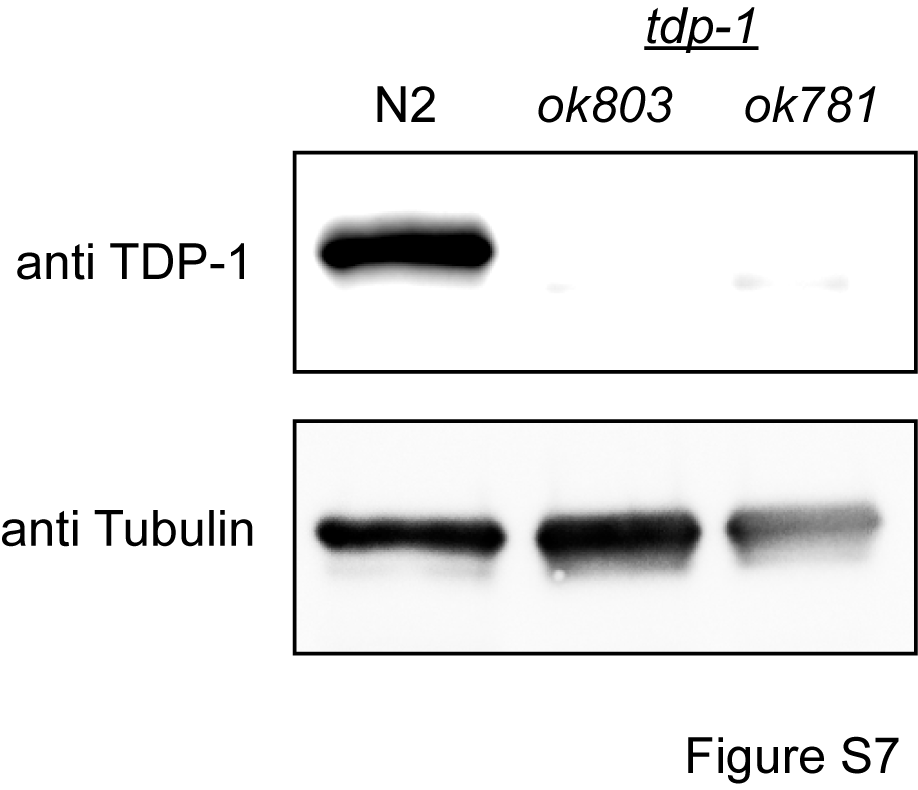

Supplement: Figure S7 — Characterization of the TDP-1 antibody. Western blotting of protein extracts from wild type N2, tdp-1(ok803) and tdp-1(ok781) strains with a polyclonal anti TDP-1 antibody revealed a signal in N2 worms but no signals from the deletion mutants ok803 or ok781. (TIF) [file pgen.1002806.s007.tif]

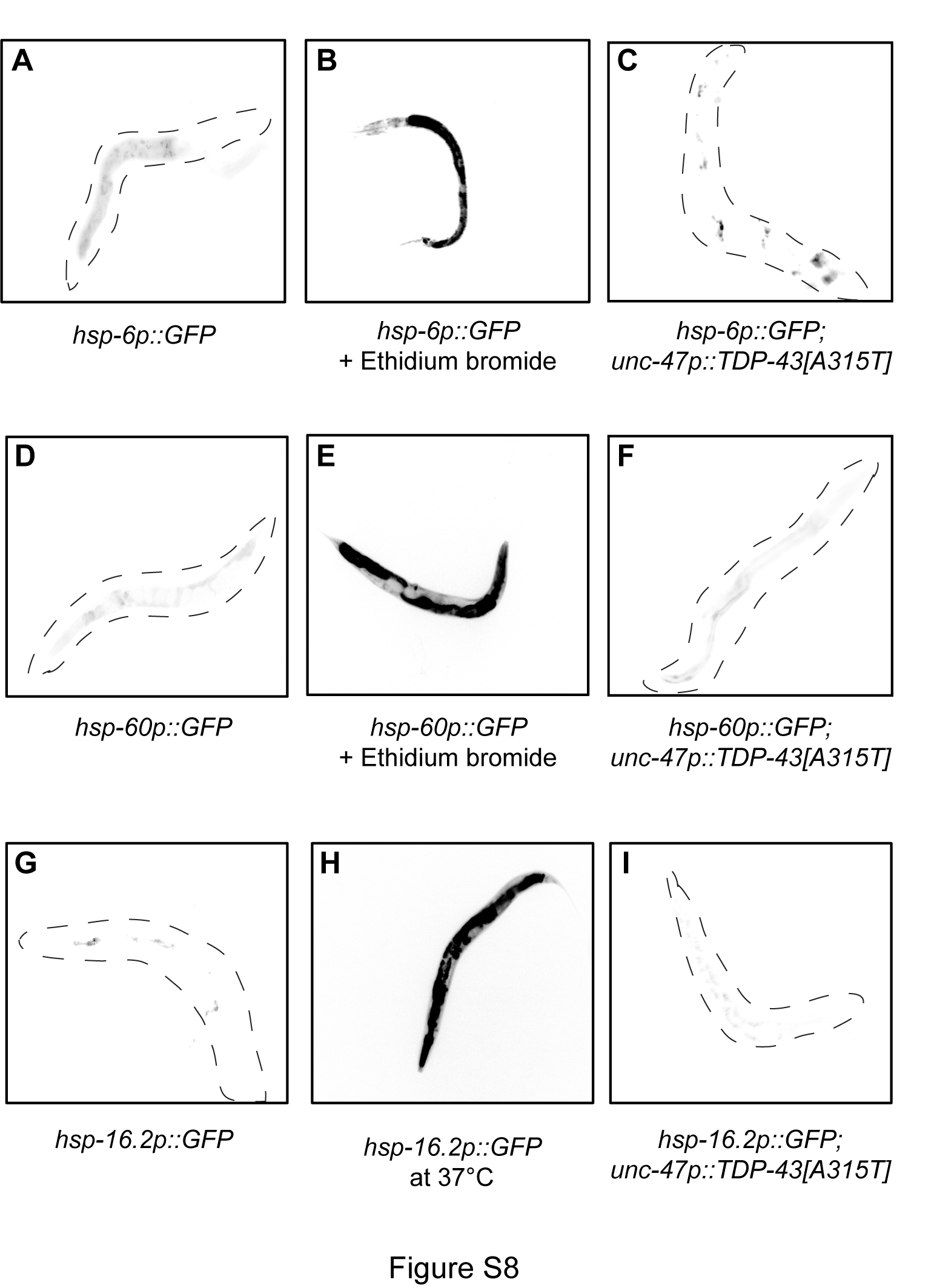

Supplement: Figure S8 — Mutant TDP-43 does not activate mitochondrial or cytoplasmic chaperones. (A–I) are low-resolution photographs of young adult transgenic worms. The images have been converted to black and white and photo-reversed to aid visualization. Difficult to see worms are outlined. (A) Low expression of the mitochondrial chaperone reporter hsp-6p::GFP under non-stress conditions. (B) Ethidium bromide induced hsp-6p::GFP expression. (C) Mutant TDP-43 did not induce hsp-6p::GFP expression. (D) Low expression of the mitochondrial chaperone reporter hsp-60p::GFP under non-stress conditions. (E) Ethidium bromide induced hsp-60p::GFP expression. (F) Mutant TDP-43 did not induce hsp-60p::GFP expression. (G) Low expression of the heat shock reporter hsp-16.2p::GFP under non-stress conditions. (H) High temperatures induced hsp-16.2p::GFP expression. (I) Mutant TDP-43 did not induce hsp-16.2p::GFP expression. (TIF) [file pgen.1002806.s008.tif]
