# Supplementary material for: TDP-1/TDP-43 Regulates Stress Signaling and Age-Dependent Proteotoxicity in Caenorhabditis elegans
Source: PLoS Genet. 2012 Jul 5;8(7):e1002806. doi: 10.1371/journal.pgen.1002806 (PMC3390363; doi:10.1371/journal.pgen.1002806)
Supplement: Table S1 — Lifespan analysis for all experiments. Related to Figure 1 and Figure 9. Animals that died prematurely (ruptured, internal hatching) or were lost (crawled off the plate) were censored at the time of scoring. All control and experimental animals were scored and transferred to new plates at the same time. n.s. not significant. (PDF) [file pgen.1002806.s009.pdf]

	Strains	Mean Life Span	p Value	75th Percentile (Days)	Maximum Lifespan	Total Number of Animals Died/Total
**Figure 1**	*daf-2(e1370)*	38		42	54	61/61
	daf-2(e1370);tdp-1(ok803)	33	<0.0001	37	41	59/60
	daf-16(mu86)	13		14	17	55/60
	daf-16(mu86) ;tdp-1(ok803)	12	n.s. 0.1205	13	17	61/62
	daf-2(e1370) + EV	41		45	53	57/63
	daf-2(e1370) + tdp-1 RNAi	28	<0.0001	37	41	54/65
	N2 (20°C)	16		21	26	57/66
	tdp-1(ok803) (20°C)	20	<0.0001	25	30	58/65
	N2 (25°C)	11		15	21	64/65
	tdp-1(ok803) (25°C)	13	n.s. 0.5768	13	21	59/59
	N2	18		22	28	49/62
	tdp-1p ::TDP-1 ::GFP	11	<0.0001	13	15	55/64
	tdp-1p ::TDP-1 ::GFP ;tdp-1(ok803)	15	<0.0001	18	21	71/77
	tdp-1(ok803)	28	<0.0001	30	31	57/62
	N2 (25°C)	12		16	22	60/65
	tdp-1p ::TDP-1 ::GFP (25°C)	08	<0.0001	10	12	60/69
**Figure 9**	*unc-47p::TDP-43[A315T]*	18		21	25	57/61
	unc-47p::TDP-43[A315T]; tdp-1p::TDP-1::GFP	15	0.0014	17	23	51/61
	unc-47p::FUS[S57Δ]	20		24	27	53/62
	unc-47p::FUS[S57Δ]; tdp-1p::TDP-1::GFP	15	<0.0001	17	21	58/62

Table S1
