# Supplementary material for: TDP-1/TDP-43 Regulates Stress Signaling and Age-Dependent Proteotoxicity in Caenorhabditis elegans
Source: PLoS Genet. 2012 Jul 5;8(7):e1002806. doi: 10.1371/journal.pgen.1002806 (PMC3390363; doi:10.1371/journal.pgen.1002806)
Supplement: Table S2 — Stress assays. Related to Figure 2 and Figure S2. Animals were examined every two hours for survival against the specified stress. (PDF) [file pgen.1002806.s010.pdf]

		Strains	Mean Life Span	p Value	75th Percentile (hours)	Maximum Lifespan	Total Number of Animals Died/Total
**Figure 2**	Juglone	N2	6		8	14	35/61
		tdp-1(ok803)	2	<0.0001	3	4	50/61
		daf-2(e1370)					01/62
		daf-2(e1370);tdp-1(ok803)	1.5	<0.0001	2	6	60/60
	H_2O_2	N2	2		4	10	61/61
		tdp-1(ok803)	1	<0.0001	1.5	10	63/63
		daf-2(e1370)	5				42/60
		daf-2(e1370);tdp-1(ok803)	1	<0.0001	1.5	4	59/60
	NaCl	N2	5		10	14	59/60
		tdp-1(ok803)	2	<0.0001	4	8	60/60
		daf-2(e1370)					10/62
		daf-2(e1370);tdp-1(ok803)		n.s. 0.4609			09/62
	Sorbitol	N2					18/66
		tdp-1(ok803)	12	<0.0001			45/63
		daf-2(e1370)					01/64
		daf-2(e1370);tdp-1(ok803)		n.s. 0.5193			02/61
**Figure S2**	Thermal	N2	7		8	10	60/60
		tdp-1(ok803)	7	n.s. 0.3545	9	12	62/62
		daf-2(e1370)	12				44/63
		daf-2(e1370);tdp-1(ok803)	12	n.s. 0.3786			43/64
	UVs	N2	12				36/59
		tdp-1(ok803)	12	n.s. 0.8520			36/60
		daf-2(e1370)					21/60
		daf-2(e1370);tdp-1(ok803)		n.s. 0.7625			19/62
	Sorbitol	N2					21/60
		tdp-1(ok803)	16	0.0026			22/60
		daf-2(e1370)					02/60
		daf-2(e1370);tdp-1(ok803)		n.s. 0.2455			05/60

Table S2
