# Supplementary material for: TDP-1/TDP-43 Regulates Stress Signaling and Age-Dependent Proteotoxicity in Caenorhabditis elegans
Source: PLoS Genet. 2012 Jul 5;8(7):e1002806. doi: 10.1371/journal.pgen.1002806 (PMC3390363; doi:10.1371/journal.pgen.1002806)
Supplement: Table S3 — Paralysis tests. Related to Figure 8 and Figure 9. (PDF) [file pgen.1002806.s011.pdf]

	Strains	p Value	Total Number of Animals Paralysed/Total
**Figure 8**	*unc-47p::TDP-43[A315T]*		65/69
	unc-47p::TDP-43[A315T]; daf-2(e1370)	<0.0001	57/63
	unc-47p::TDP-43[A315T]		65/69
	unc-47p::TDP-43[A315T]; daf-2(e1368)	<0.0001	23/63
**Figure 9**	*unc-47p::TDP-43[A315T]*		58/69
	unc-47p::TDP-43[A315T]; tdp-1p::TDP-1::GFP	<0.0001	57/60
	unc-47p::FUS[S57Δ]		51/65
	unc-47p::FUS[S57Δ]; tdp-1p::TDP-1::GFP	<0.0001	64/65
	unc-47p::TDP-43[A315T]		58/69
	unc-47p::TDP-43[A315T]; tdp-1(ok803)	<0.0001	05/63
	unc-47p::FUS[S57Δ]		51/61
	unc-47p::FUS[S57Δ]; tdp-1(ok803)	<0.0001	14/63

Table S3.
