# Supplementary material for: TDP-1/TDP-43 Regulates Stress Signaling and Age-Dependent Proteotoxicity in Caenorhabditis elegans
Source: PLoS Genet. 2012 Jul 5;8(7):e1002806. doi: 10.1371/journal.pgen.1002806 (PMC3390363; doi:10.1371/journal.pgen.1002806)
Supplement: Table S4 — Lifespan and stress assays for tdp-1(ok781). Related to Figure S3. Animals that died prematurely (ruptured, internal hatching) or were lost (crawled off the plate) were censored at the time of scoring. All control and experimental animals were scored and transferred to new plates at the same time. For the stress assays, animals were examined every two hours for survival against the specified stress. n.s. not significant. (PDF) [file pgen.1002806.s012.pdf]

		Strains	Mean Life Span	p Value	75th Percentile (hours)	Maximum Lifespan	Total Number of Animals Died/Total
**Figure S3**	Lifespan	N2	16		18	24	51/62
		tdp-1(ok781)	18	0.0002	21	28	87/96
	Juglone	N2	6		8	14	62/65
		tdp-1(ok781)	4	n.s. 0.2244	8	14	58/59
	NaCl	N2	8		11	14	63/66
		tdp-1(ok781)	8	n.s. 0.5553	10	12	64/68
	Thermal	N2	12				45/60
		tdp-1(ok781)	12	n.s. 0.5554			47/65

Table S4
